# Supplementary material for: Understanding parents’ decision-making on participation in clinical trials in children’s heart surgery: a qualitative study
Source: BMJ Open. 2021 Feb 23;11(2):e044896. doi: 10.1136/bmjopen-2020-044896 (PMC7907877; doi:10.1136/bmjopen-2020-044896)
Supplement: Supplementary data [file bmjopen-2020-044896supp001.pdf]

Drury NE et al. Understanding parents' decision-making on participation in clinical trials.

SUPPLEMENTARY MATERIAL

Table S1. Participant demographics

**Interview**	Parent(s) interviewed	Parent(s) age (years)	Parent(s) ethnicity	Type of surgery
**C1**	Mother	25-34	White British	TOF repair
**C2**	Mother	<25	White British	VSD closure
**C3**	Mother	≥35	Other	VSD closure
**C4**	Mother & Father	Both ≥35	Both South Asian	TOF repair
**C5**	Father	25-34	White British	TOF repair
**C6**	Mother	25-34	White British	TOF repair
**C7**	Mother	25-34	Black	TOF repair
**C8**	Father	25-34	White British	VSD closure
**C9**	Mother	≥35	White British	TOF repair
**C10**	Mother	25-34	White British	TOF repair
**C11**	Mother	25-34	White British	VSD closure
**C12**	Mother	25-34	South Asian	VSD closure
**C13**	Mother	≥35	White British	VSD closure
**C14**	Mother	25-34	White British	TOF repair
**C15**	Mother	25-34	White British	VSD closure
**C16**	Mother	≥35	White British	VSD closure
**C17**	Mother	<25	White British	VSD closure
**C18**	Mother & Father	Both <25	Both White British	VSD closure
**C19**	Mother	25-34	White British	TOF repair
**C20**	Mother	25-34	White British	TOF repair
**C21**	Mother & Father	Both ≥35	Both White other	VSD closure
**D1**	Mother	25-34	White British	TOF repair
**D2**	Mother	≥35	South Asian	TOF repair

C, consenting parent; D, declining parent; TOF, tetralogy of Fallot; VSD, ventricular septal defect.

Drury NE, *et al. BMJ Open* 2021; 11:e044896. doi: 10.1136/bmjopen-2020-044896

Supplementary results: The parents/guardians of 46 children were approached about their child's participation in the trial, of whom 38 consented to the trial and eight declined, as shown in the flow diagram (figure 1). Of these, six consenting parents and four declining parents declined participation in the interview sub-study and a further six consenting (four missed prior to discharge, one had learning difficulties, and one did not speak sufficient English) and two declining (one with surgical complications and one where social services had parental responsibility) parents were not recruited. Thirty-two parents of 26 children gave consent to participate in an interview but two parents withdrew consent prior to the interview, one did not attend the interview, and three were lost to follow up. We therefore interviewed 26 parents of 23 children, 24 consenting parents (of 21 children) and two declining parents (of 2 children). These comprised 21 mothers and five fathers, with three interviewed as pairs and the remainder as one-to-one interviews; child and participant-level descriptions are shown in table S1. In the quotes, C indicates a consenting parent and D signifies a declining parent.

1. Risks of participation

Risk

"*I think again it's the risk of it, I mean he's my baby, I want to make sure that it's going to be okay, it's not going to be too risky, I wouldn't put him through anything that was going to be risky.*" (C17)

"*We were taking into consideration what the research was trying to achieve and there was actually no risk, or minimal risk.*" (C21 father)

"*I think the fact that there could've been a benefit and there not being a risk made me go for it. So, had we had known 'yes there's a benefit, there'll definitely be a benefit, but there is a risk' I wouldn't have done it. It was all about the risk factor.*" (C10)

"*If there was a risk that it could've hurt him or affected the surgery, then I wouldn't have done it.*" (C1)

"*We just really wanted to know what the risks were, whether there could be any damage to her afterwards.*" ["So, for you it was just all about the risk?"] "*Yeah definitely*" (C16)

"*Well if it had no risk, or low risk shall we say, and it gives him a better chance that you know he can do a full recovery then I can't see why not. I can't see why someone wouldn't want to do it.*" (C11)

"*For me personally as long as there was no risk involvement for [child], I'm more than happy for him to participate and … use his situation to help with your research. So yeah as long as there's no actual risk.*" (C19)

"*Yes absolutely, definitely, 100%. If there were any risks involved, then we wouldn't have been so - we would have to have sat down and thought about it and maybe had more conversations with whoever was leading it… It was just that there was no risk. That was the biggest thing for us.*" (C6)

"*Again, it always boils down to risk doesn't it, side effects. I think that would always be the big question for me because I wouldn't put my child under any more duress or stress than what she'd need to be.*" (C16)

<u>Additional procedures</u>

"*I suppose because it wasn't anything invasive, I was more inclined to say, 'Yes, just do it.' It wasn't going to affect her in any way whatsoever, and so why not? Let somebody else benefit from the results. If it was invasive, I would obviously have thought, 'Let's look into this.'*" (C20)

"*…because she already had lines in and they said, 'I just need to take a bit of blood', I said, 'Yeah, knock yourself out.* [laughter] *It's not harming her. It's not doing anything'… She didn't have a clue what was going on, bless her.*" (C20)

"*So, it was a case of them just taking extra samples or whatever the case may be, so we didn't really have to do anything.*" (C19)

"*I might have been a little bit more worried then because she was getting upset … so if that would have been the case I probably would have said no, because I wouldn't have wanted to have upset her any more, but because it was in, then yes, I was quite happy.*" (C16)

"*I think just making sure that there was no extra needles and cannulas put in, otherwise everything was fine for me to go ahead.*" (C7)

"*Yeah, I think I'd probably say no because she was traumatised when they did it. Every time they did it in there, she was traumatised.*" (C13)

"*He got all burst blood vessels from crying from the last time they pricked him. No, I wouldn't have allowed it – sorry.*" (C21 mother)

"*I'm very much that you can't progress and learn more if people don't get involved in things like research. Whereas my wife was, and me to a certain extent, was very much like she's*

already been through enough, without putting her through anything else while she's having surgery." (C8)

"*For weeks before we were told not to put her through anything over what she was going through already, like to really watch when she was crying, she's too sweaty. Everything was like, don't let her go. And then to have somebody say, well we're going to do this as extra, we're like well hang on, does she really need that on top of everything else?*" (C8)

Change of mind

"*I kind of feel bad now… I think I'd say yes to be honest. Now I know it's only that and not part of the operation… now I've been through it, I know obviously it's stressing and they get annoyed with everything anyway, so another thing isn't going to harm her.*" (D1)

2. Personal benefit and altruism

Personal benefit

"*It might not help my daughter this time, but research… might help her in the future.*" (C6)

"*I think reducing lifelong risks, such as damage… she might have to go and have another surgery when she's 20. I'm hoping with the research from now until then, it might not be open-heart surgery; it might be something very, very non-invasive and that's when I think the research will come into play.*" (C6)

"*Anything that we can do to help which might help her in the long run is definitely, we will do.*" (C19)

"*Our first baby, she's had tetralogy of Fallot, so automatically our percentage goes up where if we have another pregnancy that that baby could have a heart condition as well. So, you're almost helping yourself a little bit by agreeing to research on the first operation because you might be there again with the second one, do you know what I mean?*" (C10)

"*You then have a baby that actually needs something and so you need to have the research for the future. I now know that there's a risk that if I have another child, there could be a heart problem. Now we've looked back on it, heart problems actually run through the family. Obviously, your research may help my baby in the future.*" (C20)

"*If anything from the research that they did have it could have positive gains, so we weren't concerned at all about that.*" (C19)

"*Obviously for her to be in the position she was in and having the surgery that she was having, people must've had research studies done on them beforehand, so I just think it helps people and babies that are in our position now, in a few years. If it can make it better for them, make it a better process for them then why not do it? If there's no risk, because there was no risk to [child] through the whole process, so.*" (C10)

Altruism

"*When we left the hospital, I said, 'Oh god, we've got to go back to the real world now.' Whereas, back there, your child has got a heart problem, but so has that child and that child. You're listening to all the different scenarios, stories and medications.*" (C20)

"*You're thinking, oh my God my child's having open heart surgery you don't want nothing extra going on. But this is the main reason why we did it because he's not the first and he's never going to be the last. So, if we didn't agree for things like this, it would never get better for kids in the future.*" (C4 mother)

"*Obviously for her to be in the position she was in and having the surgery that she was having, people must've had research studies done on them beforehand, so I just think it helps people and babies that are in our position now, in a few years. If it can make it better for them, make it a better process for them then why not do it?*" (C10)

"*I feel good about it if it can help somebody. I feel good about it anyway because I feel like it's trying to help… She sure is doing some good in the world… If we can do anything to help any children, then we're willing to do it.*" (C13)

"*I feel lucky that we got a chance to take part in it. Because if the end result is something positive then that's brilliant for future babies and children that have congenital heart defects.*" (C10)

"*You feel a bit privileged really don't you when you're getting chosen to do something.*" (C2)

"*I just thought she might help other babies in the future go through a better process of having their hearts fixed, long term.*" (C10)

["So, when you agreed for [child] to take part in the study, what did you hope it would achieve?"] "*Just helping other people. I think helping other people in similar situations.*" (C7)

"*I'm happy that we did it, I'm happy that she got to take part in something that might change the way that heart patients are treated in the future.*" (C10)

"*It wasn't going to affect her in any way whatsoever, and so why not? Let somebody else benefit from the results.*" (C20)

"*Yeah quite happy to, I mean it was all for the greater good isn't it... it's actually helping future families like yourselves and their children… It's a good thing, it's the only way we're going to learn isn't it so if we can help and be part of that then yeah, we're happy to do our bit.*" (C19)

3. Information, understanding and timing

<u>Information</u>

"*It did give us time to ponder over it and you know look things up on the internet or speak to people. I think if we hadn't had that opportunity, I think we would have declined it.*" (C16)

"*Explain in the letter. Explain in the phone call. When you send that letter out, say, 'Please contact us if you wish to.' They can then contact you or you get the phone call in, so that you can explain the process afterwards as well. You can then say, 'Right, this is what it's actually going to entail from start to finish.*" (C20)

<u>Understanding</u>

"*Obviously, they were looking at, was it the blood pressure reducing up and down, so that it's not as tiring for the heart? I think I've got that from somewhere.*" (C14)

"*It's something to do with stopping blood flow to the heart before you pop it on bypass, and I can't remember… Is it to do with like how the hearts survives heart attacks, like cardiac arrests and stuff like that?*" (C10)

["Did you understand what the study was trying to find out?"] "*I can't remember that much!*" [laughs] (C7)

"*As far as I was aware it was going to, the cuffs were going to inflate and deflate and it was going to see whether something was released into her blood that was going to help the heart, that was kind of how it was explained.*" (C8)

"*Just that he was picked, I don't know really, it was just he was either going to be chosen or he wasn't for the study.*" (C2)

"*Picking people at random who've obviously all got a heart condition, but it might be something different to what* [she] *had.*" (C5)

Timing

"*Reading through that paperwork is the biggest thing, trying to process it because it's okay people telling you... but when you read it you think over it better yourself… The more information you get, the calmer you feel really.*" (C2)

"*I'd like to speak to the surgeons and ask more questions from them as to any concerns, rather than just getting a sheet of information because you always have extra questions, I'd want to ask a few more.*" (C19)

"*Having the time and people to help you understand what's going to happen, so you fully understand what's going to happen, because we didn't fully understand.*" (C8)

"*The Friday before she was due to go in, we came and spoke to the surgeons and we came and spoke to a nurse and that's the time when they talk you through the operation and things. So, any questions we had about that research we could have covered then.*" (C8)

"*We did ask for advice. In that respect, perhaps if it would have been rushed or an intense environment, then it would have been a bit harder for us to come to that decision.*" (C21 father)

"*I mean we had just enough time I think, we had about a week from when we got the letter to when we actually got the phone call to come in. So that wasn't too bad, that was enough time for me.*" (C17)

"*I think to have to make that decision in a short space of time, some people can, because they can cope with it, me personally I couldn't. I couldn't just make that decision because I'd just be thinking to get my child medically well and I'd probably be thinking a research study is the last thing on my mind to be dealing with.*" (C16)

"*And it's a lot to take in then when you're also thinking you're going to be having an operation, and then there's something else you're being asked to do… obviously from a parent perspective anything is unnerving anyway, your child's going through that as it is, you don't want to add anything else.*" (D1)

"*No, because it's just one of those things we don't need to worry about when you've got so much in our head as it is… when you are going through something, only you know what you are going through and at that point you can't even think straight.*" (D2)

"*I think if it was an emergency situation maybe my decision may not have been so quick or whether it would happen at all, really.*" (C6)

"*If you're approached just days before the operation, you might not want to do it. But if you're approached long before you're even given a date, as long as you've got it in your mind that something's taking place, I think more and more people would do it.*" (C4 mother)

"*Well, obviously when you gave the phone call it was a lot more, I'd got a bit of time to think about whether or not I want him to take part. So, with them asking me on the phone and then when we actually got there then speaking to us properly about it and trying to explain everything it was a lot more easier than somebody just coming into your room and saying, 'Well this is what we need to do, he's going for his operation in like two hours so can you decide on whether you want him to do it or not?*'" (C2)

"*I think, it all goes back to time. I think if we'd had more time to read it and we were less emotional so we weren't actually in the hospital waiting and had the opportunity to speak to someone about it at the point when we came to have a look around ICU… we could have had someone sit down and just spend five minutes explaining it. Because when we talked about the heart, they got a little model out and they explained exactly what they were going to do, exactly what the risks were. I think something similar to that would have helped.*" (C8)

"*At that point I was lost* [on intensive care]. *My baby was in heart failure because he was late diagnosed and if things were different, like I said, I panic and I hit the panic button quite quickly so for a mum like me I don't think I'd be listening to anyone.*" (C3)

"*When you're sitting there and like when we first walked in and saw* [daughter] *tubed up and machines breathing for her, it really did hit home what she'd been through. But I think as the days go by and you're sitting there, I think you do become more relaxed when they start turning machines off so, if someone came to you after a couple of days saying, 'Do you mind if we just try this?'*" (C5)

"*It might have been a bit overwhelming because the research thing is an extra and sometimes, parents don't have the ability to think of these extra things when they're in an Intensive Care environment.*" (C21 mother)

"*Next to us, in PICU, was a very young baby. My wife spoke to the parents and they didn't know that their baby had a heart condition. I think they got rushed into the hospital and then it was one surgery after the other. They were quite overwhelmed. They were very receptive parents but in such a situation, I don't know how much they would factor in that there were studies going on in ICU or in the hospital.*" (C21 father)

4. Trust in the clinical team

"*We just had a great deal of confidence in Birmingham Children's* [Hospital]*. They really instilled us with so much confidence.*" (C6)

"*You just have to listen to what the doctors are telling you and put your trust in them that they know what they're doing, they're the experts.*" (C16)

"*I thought to myself that surgeons know best, if it was going to affect her in any way, I don't think they'd agree to it generally. So I guess it's just putting that trust in the surgeon, they know what's better for the patient when they open them up… so if the surgeon felt that it wasn't appropriate… they probably would have said no thank you.*" (C12)

"*You just trust the surgeons that they take as much or as little as they need to, and they do… I suppose we just put our trust in them that they're doing what they need to do.*" (C19)

"*I'll put my trust in the NHS and I know that they know what they're doing, and I wouldn't question like…*" (C18 mother)

"*If you've got these exceptional surgeons and doctors telling you, 'This is serious, but we need to do it now, ready for when she's older,' you take that information… It was purely, we didn't have to think, 'Oh, we don't want the surgery.' We didn't have to think because it was just so absolutely, 'Get her in as soon as you can,' really. I wanted it done as soon as possible.*" (C6)

"*When he came out and he was looking at her, he actually did look almost proud of his work. That fills you with confidence because you think, 'If you think that's good...' I don't know, I'm not a surgeon… When we were sat there and he looked proud of his work, I thought, 'Okay, yeah. We're good. We're alright. We're in safe hands.'* [Laughter] *It's all about being safe, isn't it?*" (C20)
