# Supplementary material for: Understanding parents’ decision-making on participation in clinical trials in children’s heart surgery: a qualitative study
Source: BMJ Open. 2021 Feb 23;11(2):e044896. doi: 10.1136/bmjopen-2020-044896 (PMC7907877; doi:10.1136/bmjopen-2020-044896)
Supplement: Author's manuscript [file bmjopen-2020-044896.draft_revisions.pdf]

BMJ Open is committed to open peer review. As part of this commitment we make the peer review history of every article we publish publicly available.

When an article is published we post the peer reviewers' comments and the authors' responses online. We also post the versions of the paper that were used during peer review. These are the versions that the peer review comments apply to.

The versions of the paper that follow are the versions that were submitted during the peer review process. They are not the versions of record or the final published versions. They should not be cited or distributed as the published version of this manuscript.

BMJ Open is an open access journal and the full, final, typeset and author-corrected version of record of the manuscript is available on our site with no access controls, subscription charges or pay-per-view fees (http://bmjopen.bmj.com).

If you have any questions on BMJ Open's open peer review process please email info.bmjopen@bmj.com

BMJ Open

Understanding parents' decision-making on participation in clinical trials in children's heart surgery: a qualitative study

Journal:	*BMJ Open*
Manuscript ID	bmjopen-2020-044896
Article Type:	Original research
Date Submitted by the Author:	16-Sep-2020

Complete List of Authors:	Drury, Nigel ; University of Birmingham, Institute of Cardiovascular Sciences; Birmingham Children's Hospital NHS Foundation Trust, Paediatric Cardiac Surgery<br>Menzies, Julie; Birmingham Children's Hospital, Paediatric Intensive Care<br>Taylor, Clare; University of Oxford, Nuffield Department of Primary Care Health Sciences<br>Jones, Timothy; Birmingham Children's Hospital, Paediatric Cardiac Surgery; University of Birmingham, Institute of Cardiovascular Sciences<br>Lavis, Anna; University of Birmingham, Institute of Applied Health Research

Keywords:	Paediatric cardiac surgery < PAEDIATRIC SURGERY, QUALITATIVE RESEARCH, Clinical trials < THERAPEUTICS

SCHOLARONE™
Manuscripts

I, the Submitting Author has the right to grant and does grant on behalf of all authors of the Work (as defined in the below author licence), an exclusive licence and/or a non-exclusive licence for contributions from authors who are: i) UK Crown employees; ii) where BMJ has agreed a CC-BY licence shall apply, and/or iii) in accordance with the terms applicable for US Federal Government officers or employees acting as part of their official duties; on a worldwide, perpetual, irrevocable, royalty-free basis to BMJ Publishing Group Ltd ("BMJ") its licensees and where the relevant Journal is co-owned by BMJ to the co-owners of the Journal, to publish the Work in this journal and any other BMJ products and to exploit all rights, as set out in our licence.

The Submitting Author accepts and understands that any supply made under these terms is made by BMJ to the Submitting Author unless you are acting as an employee on behalf of your employer or a postgraduate student of an affiliated institution which is paying any applicable article publishing charge ("APC") for Open Access articles. Where the Submitting Author wishes to make the Work available on an Open Access basis (and intends to pay the relevant APC), the terms of reuse of such Open Access shall be governed by a Creative Commons licence – details of these licences and which Creative Commons licence will apply to this Work are set out in our licence referred to above.

Other than as permitted in any relevant BMJ Author's Self Archiving Policies, I confirm this Work has not been accepted for publication elsewhere, is not being considered for publication elsewhere and does not duplicate material already published. I confirm all authors consent to publication of this Work and authorise the granting of this licence.

For peer review only

Understanding parents' decision-making on participation in clinical trials in children's heart surgery: a qualitative study

Nigel E Drury [1,3*], Julie C Menzies [2*], Clare J Taylor [5], Timothy J Jones [1,3], Anna C Lavis [4]

[1] Department of Paediatric Cardiac Surgery, Birmingham Children's Hospital, Birmingham, UK; [2] Department of Paediatric Intensive Care, Birmingham Children's Hospital, Birmingham, UK; [3] Institute of Cardiovascular Sciences, University of Birmingham, Birmingham, UK; [4] Institute of Applied Health Research, University of Birmingham, Birmingham, UK; [5] Nuffield Department of Primary Care Health Sciences, University of Oxford, Oxford, UK.

* Nigel Drury and Julie Menzies contributed equally to this work.

Correspondence: Mr Nigel Drury, Department of Paediatric Cardiac Surgery, Birmingham Children's Hospital, Steelhouse Lane, Birmingham, B4 6NH, UK.

Tel: +44 121 333 8731, fax: +44 121 333 9441, email: n.e.drury@bham.ac.uk

Word count: 3892 (excluding title page, abstract, references, figures, and table)

Number of figures: 2

Number of tables: 1

Keywords: Paediatric cardiac surgery, parents, qualitative study, clinical trials, decision-making.

For peer review only - http://bmjopen.bmj.com/site/about/guidelines.xhtml

Abstract

Objectives: Few children undergoing heart surgery are recruited to clinical trials and little is known about the views and attitudes of parents towards trials. This study explored parents' perspectives on decision-making about their child's participation in a clinical trial during their elective cardiac surgery.

Design: Qualitative interview study.

Setting: Single centre sub-study of a multi-centre, double-blind, randomised controlled trial to investigate the effects of remote ischaemic preconditioning in children undergoing cardiac surgery.

Participants: Parents of children approached to participate in the trial, both consenters and decliners.

Methods: Semi-structured interviews were conducted face-to-face or by telephone following discharge, digitally audio-recorded, transcribed, and thematically analysed.

Results: Of 46 patients approached for the trial, 24 consenting and 2 declining parents agreed to participate in an interview (21 mothers, 5 fathers). Parental decision-making about research was influenced by 1) potential risks or additional procedures, 2) personal benefit and altruism for the 'cardiac community'; 3) information, preparation, timing and approach; and 4) trust in the clinical team and collaboration with researchers. All of these were placed within the context of their understanding of the trial and knowledge of research.

Conclusions: Parents of children undergoing cardiac surgery attach value to clinical research and are supportive of clinical trials when there is no or minimal perceived additional risk. These findings enhance our understanding of the factors that influence parents' decision-making and should be used to inform the design and conduct of future paediatric surgical trials.

Abstract word count: 238

For peer review only

For peer review only - http://bmjopen.bmj.com/site/about/guidelines.xhtml

Strengths and limitations of this study

- This qualitative sub-study aimed to identify the most important issues that influence parents' decision-making on whether to allow their child to take part in a surgical trial, to improve the design and conduct of future trials.
- The interview topic guide, protocol, and study documents were developed with extensive patient and public involvement.
- The clinical trial was a suitable vehicle to explore parents' perspectives on research as the surgery was elective with low predicted mortality and the trial intervention presented minimal additional risk.
- The study reached data saturation for parents who consented to the trial but was limited by the low number of parents who agreed to be interviewed after declining their child's participation.
- Parents were recruited from a single large paediatric cardiac surgical centre in the United Kingdom which may limit generalisability.

For peer review only

INTRODUCTION

Randomised controlled trials are the accepted gold standard to evaluate the efficacy of treatments, promote evidence-based practice and improve the quality of clinical care. However, of the approximately 4,500 children who undergo surgery for congenital heart disease annually in the United Kingdom [1], less than 1% have been recruited to cardiac surgical trials, all of which have been small, single-centre, phase II trials [2]; in contrast, over 70% of children diagnosed with cancer are enrolled into national or international late phase trials [3] As a congenital heart disease community, we have a responsibility to conduct well-designed, multi-centre trials to answer key questions to improve the outcomes of surgery for children and their families [2].

Recruitment to paediatric trials is recognised to be challenging [4,5] but can be improved by understanding the factors that are important to parents when considering whether to allow their child to take part. The role of parents in this decision-making is complex, balancing the perceived risks and benefits of taking part [6]. However, little is known about the views and attitudes of the parents of children undergoing cardiac surgery towards involvement in research and specifically clinical trials; by understanding parents' perspectives, we can support their decision-making by improving the design and conduct of future trials. This knowledge is useful for enhancing families' experiences of trial participation and increasing recruitment [6], thereby expanding the evidence-base to guide treatment and improve patient outcomes. Qualitative studies provide participants' experiences in their own words, allowing exploration of the meanings they attribute to them, which is crucial to getting beyond assumptions about what matters in the processes of decision-making [7]. We conducted interviews with the parents of children approached to participate in a low-risk, double-blind, randomised controlled trial to explore their perspectives on research involving their child, with the aim to better understand the factors that influence their decision whether or not to participate in a clinical trial.

For peer review only - http://bmjopen.bmj.com/site/about/guidelines.xhtml

MATERIALS AND METHODS

This qualitative study was a single-centre sub-study of the Bilateral Remote Ischaemic Conditioning in Children (BRICC) trial, a multi-centre, double-blind, randomised controlled trial of remote ischaemic preconditioning in children (ISRCTN 12923441) [8]. In the trial, children aged 3 months to 3 years undergoing elective surgery for either isolated ventricular septal defect closure or tetralogy of Fallot repair were recruited. Bilateral lower limb preconditioning was performed after induction of anaesthesia but prior to sternotomy. Right atrial (additional) +/- right ventricular (when routinely resected) biopsies were obtained intraoperatively and blood samples were taken from indwelling lines during the first 24 hours after surgery. Both the clinical trial (16/WM/0309, 5 August 2016) and this qualitative sub-study (17/WM/088, 28 April 2017) were approved by the West Midlands-Solihull NHS Research Ethics Committee and sponsored by the University of Birmingham (RG_14-025 and RG_17-005, respectively).

Patient and Public Involvement (PPI): The sub-study protocol was reviewed and amended following feedback from the Clinical Research Network's Young Person's Steering Group in the West Midlands, comprising 11 young people and one parent. Four parents of children who had previously undergone cardiac surgery reviewed the parent/guardian information sheets (PIS) and consent forms to improve clarity and readability. Another eight parents were convened as a focus group (facilitated by NED and ACL) to discuss their opinions, beliefs, concerns and expectations of research in children's heart surgery [9], and this was used to develop an interview topic guide.

Participants: Parents of children approached to participate in the trial, both those who consented and those who declined, were eligible to be interviewed. Potential participants were excluded if their child had experienced a serious adverse event during or immediately after surgery to avoid further distress, including death, extracorporeal life support or further surgery in the early post-operative period, or if their level of English was insufficient to

For peer review only - http://bmjopen.bmj.com/site/about/guidelines.xhtml

participate in the interview process. Recruitment began six months after starting the trial to allow time for the healthcare professionals to become familiar with the trial processes. Subsequently, all eligible parents were approached to participate, and it was estimated that the parents of 20-30 children would be interviewed but recruitment would stop if data saturation were reached or trial recruitment was completed.

Recruitment: Following discharge from PICU, one or both parents were approached on the ward by a research nurse. Parents were provided with the PIS and offered the opportunity to participate in an interview at a convenient time: prior to discharge, face-to-face at home, at an outpatient visit, or by telephone. With written informed consent, semi-structured interviews were conducted within one month of discharge by an experienced qualitative interviewer (JCM), previously unknown to the parents and independent of the clinical team.

Interviews: The schedule included: understanding of the trial, research knowledge, provision of information, timing of approach, acceptability, motivations for participation, types of research, and most important factors in decision-making. As subsequent member checking by participants for accuracy and resonance with their experiences was deemed impractical, participant validation was performed during the interviews by summarising, repeating or paraphrasing the participants' words [7]. Field notes were recorded to provide context, aid interpretation, and document emotions and non-verbal behaviours. Risk to participants was deemed to be minimal but in the event of distress or concern about events which had occurred, parents were signposted to their cardiac specialist nurse, General Practitioner, or the Patient Advice Liaison Service (PALS), as appropriate.

Analysis: Interviews were digitally audio-recorded, professionally transcribed, anonymised, and thematically analysed [10] by three researchers (JCM, NED, ACL), using NVivo 12 (QSR International, Melbourne, Australia) for data management. The initial eight interviews were coded by all researchers independently, the coding structure compared to ensure consistency and a common coding scheme developed. The remaining interviews were

For peer review only - http://bmjopen.bmj.com/site/about/guidelines.xhtml

allotted between the three researchers who coded these independently and convened to generate themes and discuss deviant cases.

The first authors had full access to all the data and take responsibility for its integrity and analysis.

For peer review only

For peer review only - http://bmjopen.bmj.com/site/about/guidelines.xhtml

RESULTS

Between September 2017 and June 2019, the parents/guardians of 46 children were approached about their child's participation in the BRICC trial at the Birmingham site, of whom 38 consented to the trial and eight declined (figure 1). Interviews were conducted with 26 parents of 23 children, 24 consenting parents (of 21 children) and two declining parents (of two children); child and participant demographics are shown in table 1, with participant-level descriptions in the supplement. Data collection was stopped after interviewing 26 parents as it was agreed that data saturation had been reached.

Parental decision-making about whether their child should participate in cardiac surgical research was influenced by four key factors: 1) risks of participation and additional procedures; 2) personal benefit and altruism for the 'cardiac community'; 3) information, understanding and timing of approach; and 4) trust in the clinical team (figure 2). These were placed within the context of their understanding of the trial and knowledge of research. In the quotes, C indicates a consenting parent and D signifies a declining parent; additional quotes are provided in the supplement.

1. Risks of participation

Parental decision-making was influenced by the perceived level of risk for potential harm posed by the research. Some parents weighed up the risks and benefits of their child participating whilst for others, the focus on any perceived risk trumped any potential benefit; they could overlook a lack of personal benefit to help others but only if there was no risk to their own child.

> "*I was quite open to it because there was no risk… If there'd been a risk to her, I wouldn't have done it.*" (C10)

For peer review only - http://bmjopen.bmj.com/site/about/guidelines.xhtml

The trial involved additional procedures to standard care, such as blood sampling on PICU and intraoperative tissue biopsies. Parents were largely unconcerned about the additional blood samples, even though these were 'extra', because samples were taken from indwelling lines and therefore not associated with painful procedures. If this had involved additional venepuncture, parental attitudes would have changed; for many, the added potential distress to their child would be the determining factor.

Both parents who declined the trial were apprehensive about being involved in research *per se*, that it is something extra and therefore has an inherent and unnecessary risk.

> "*They wanted to know if it would make things easier with the operation but for us, we thought they were experimenting, so we didn't want it done. Just in case something went wrong whilst they did it. I know it's just a cuff but it's just one of those things.*" (D2)

Although, on reflection during the interview, one parent (D1) had changed her mind and would now be willing to take part.

2. Personal benefit and altruism

Parents often described considering personal benefit for their child in their decision-making. They recognised that whilst the trial intervention may have a direct benefit, the research may also help their child later in life, especially if they required further surgery. Several parents considered the potential benefit to any future unborn children who may also have a congenital heart defect and require surgery.

> "*You need to have the research for the future. I now know that there's a risk that if I have another child, there could be a heart problem. Now we've looked back on it, heart problems actually run in our family… research may help my baby in the future.*" (C20)

For peer review only - http://bmjopen.bmj.com/site/about/guidelines.xhtml

Families felt a strong sense of being part of a community affected by congenital heart disease, particularly whilst in hospital. Many reflected that they were benefitting from previous parents' participation in research and that they could contribute to improving surgery and outcomes for future families. They were positive about how participating in the trial made them feel and satisfaction that their child had contributed to research. Parents who had declined the trial also recognised that participation in research may benefit other children but focused on the needs of their own child.

> "*Obviously, it's not like you don't care about anyone else's child but you know when you are going through it it's like you want everything the best for them.*" (D2)

3. Information, understanding and timing

Parents described the turmoil of getting their heads around the cardiac diagnosis, what the surgery involved and how it would impact on them and their child, and it was against this background that decisions about trial participation were made. The trial PIS was written with PPI input and most parents found the quantity and complexity of information to be appropriate.

> "*It was explained well, it wasn't full of, you know, big words that you don't understand and equally you know, yeah I do feel it was aimed at the right audience really.*" (C16)

However, there were often misconceptions about the trial, the intervention and how it related to the operation. There was a lack of confidence in, and some misunderstanding of, research terminology such as 'randomisation', 'blinding' and 'placebo'. Misconceptions involved a belief that their child was 'chosen' for the study or not understanding that the study had set inclusion and exclusion criteria.

For peer review only - http://bmjopen.bmj.com/site/about/guidelines.xhtml

Most parents had been sent the information by post to give them time to consider their child's involvement and discuss with family members; most found this approach agreeable and valued the time to read and reflect.

> "*We probably didn't read it as well as we could have, but we did screenshot the information and send it to family members and asked them their opinion, so they had time and opportunity to read it without the emotion that we were going through.*" (C8)

Having had the opportunity to ask questions was also important, either in the clinic or on admission to the ward. When asked about how they would feel if they had to make an urgent decision, perhaps if their child was on PICU or the surgery was imminent, there was much greater apprehension.

> "*I think the time issue is very important for people. If it was an emergency and their child is having surgery, they probably haven't digested that and then to receive notification about this it might be overwhelming for some people and it might have been overwhelming for us too, really.*" (C6)

The key reason given by both parents who declined the trial was that they felt overwhelmed by their situation, with the forthcoming operation and their responsibilities for the rest of their families; they saw the research as an additional burden on their time, energy and emotions.

4. Trust in the clinical team

Families had a high level of confidence and trust in their clinical caregivers; the interviews were full of positivity about their surgeon, the wider clinical team and the National Health Service (NHS). Positive relationships between researchers and clinicians were associated with favourable perceptions of research and acceptability. Even when they did not understand the research or concepts such as randomisation, as noted above, some parents felt that their understanding, or lack of, was not important, because of their trust that the

For peer review only - http://bmjopen.bmj.com/site/about/guidelines.xhtml

For peer review only

clinical team would not do anything to harm their child. On the one hand, this meant that they did not mind if they were blinded:

> "*It doesn't really bother me to be fair because obviously you know what you're doing, and you know what…*" (C18 father) "*He's in the right hands and we trust them completely.*" (C18 mother)

whilst on the other, it suggests that there is some misunderstanding about the role of research and its relationship with clinical practice. This links back to the concepts of personal risk and benefit, in suggesting that parents may not perceive the depersonalised aspects of research, such as randomisation, instead trusting that even in the context of a trial, the surgeon will 'choose' to do what is 'best' for their child.

For peer review only

DISCUSSION

The diagnosis of congenital heart disease and the realisation that their child requires surgery is a particularly stressful time for parents [11]. In other conditions, the seriousness of the child's disease, risk of the intervention and urgency of participation have been identified as important influences on how parents experience recruitment, their sense of vulnerability and the success of communication [12]; however, few studies have explored parents' perspectives on clinical trials involving their child undergoing heart surgery. Hoehn et al analysed the unsolicited comments of parents of neonates undergoing cardiac surgery in Philadelphia, PA regarding their reasons for agreeing or declining to participate in research; the most common reasons were societal benefit, individual benefit for their child and perception that it posed no harm, although parents also expressed concern about the risk of the study and anti-experimentation views [13]. The same group evaluated parental decision-making using a competence assessment tool and found that despite the stress of surgery, parents were able to understand study-specific information and make informed decisions on their neonate's participation in research [14]. Finally, Hoffman and colleagues surveyed parents of children admitted for elective cardiac surgery in Columbus, OH and found that 91% thought that clinical trials would improve the quality of care whilst 74% believed that their child may receive direct benefit from enrolling in a trial [15]. In the present study, we explored the factors that influence parents' decision-making on their child's participation in a cardiac surgical trial and identified four key aspects relating to perceived risk, potential benefit to their child or others, information and timing of approach, and trust in the clinical team.

Risk

As surrogate decision-makers, the overriding consideration of parents is to act in the best interests of their child and protect them from harm [6]. Parents often feel responsible for their

For peer review only - http://bmjopen.bmj.com/site/about/guidelines.xhtml

child's outcome in a trial and find giving consent for the child to participate much more difficult than if they were participating themselves [4]. This decision may be made more difficult by the complexity of the information and the uncertainties inherent to clinical trials, including group allocation and potential benefit from the intervention; the concepts of randomisation and clinical equipoise may be both cognitively and emotionally challenging, raising concern that their child may receive a less effective treatment [16]. The sense of responsibility for decision-making may also make parents more vulnerable to regret over making the 'wrong' decision, whether they agree to participate or not, and anticipation of regret for 'failing to protect' their child may be a major influence [6]. We found that most parents were reassured that the intervention posed no or minimal additional risk and were happy for extra blood samples to be taken if it did not negatively impact on their child. Not wanting anything extra done has been identified as a common reason for declining participation [17], but by only obtaining blood from indwelling catheters, we avoided any additional pain or distress.

Benefit

Most parents find research a positive experience and are motivated by feelings of 'doing something important' and 'giving something back' [18]. Numerous studies have identified altruism as an important factor in paediatric trials, independent of any potential personal benefit to their own child [13,19-21]. We found that parents felt motivated by a strong sense of belonging to a community affected by congenital heart disease who may benefit from the research. They also recognised that their child or their siblings may directly benefit from the research if they needed future surgery; parents of children with tetralogy of Fallot are counselled that there is a high chance of needing further surgery in early adult life so they may feel more invested in the advances that future research may bring.

For peer review only - http://bmjopen.bmj.com/site/about/guidelines.xhtml

Information, understanding and timing

Parents are more likely to allow their child to participate if they have a greater understanding of the specific study and broader trial concepts [19,22]. The use of PPI in refining the clarity and readability of written trial information has been shown to improve understanding [23] whilst educational resources, such as the *Children and Clinical Studies* programme [24], can improve parental comprehension of clinical trials and be a valuable tool to aid decision-making [25].

In the setting of elective surgery, sending out the PIS in the post provided more time for parents to consider the research and an opportunity to discuss with others, including family members and their healthcare providers [19]. Parents are more likely to take part in a trial if they feel less time pressured [22]. When asked about potentially time-critical decision-making, they were far less certain about participating, even if they were very supportive of research. Although based on projection rather than lived experience, for many, the default position would be to decline; feeling overwhelmed has been identified as the most common reason for declining participation in non-therapeutic trials on PICU [17] and parents of children undergoing cardiac surgery may be less likely to consent that other parents [26]. This suggests that recruitment involving neonates undergoing surgery in the first few days of life may be more difficult, although the 84% recruitment rate for the parents of neonates undergoing the Norwood operation in the Single Ventricle Reconstruction trial is reassuring [27]. In this group, recruitment may be optimised by prenatal trial counselling, early provision of the PIS and frequent communication.

Trust

Parents' trust in the health care professionals looking after their child may influence their decision-making [19]. In this study, parents reported immense confidence in the whole multi-disciplinary team and as found by others [15], preferred for a trial to be explained by their

For peer review only - http://bmjopen.bmj.com/site/about/guidelines.xhtml

doctor or surgeon to provide reassurance on its validity and appropriateness for their child. If this is not possible, the Principal Investigator or research coordinator should be introduced by the clinical team; a close and visible working relationship between researchers and clinicians may make recruitment more effective, building on the family's trust and respect for their healthcare provider [5] and reducing inappropriate or poorly timed approaches [20]. For some, that trust extended further, outweighing their need to understand the study, believing that the clinical team would only do 'what is best' for their child.

Declining the trial

Previous studies have identified many reasons for parents declining consent including perceived risk, pain or distress, interference with routine care, child's clinical condition, avoiding additional medications or a placebo, parental anxiety, time pressure, inadequate information or understanding, lack of importance, inconvenience, approached for too many studies, and anti-experimentalism [13,17,20,22]. Both of our decliners referred to the trial as 'just another thing to worry about' and one expressed anti-experimentation views. Identifying ways to address these issues may improve recruitment to future trials.

How can we improve clinical trials in children?

Our findings suggest several factors which should be considered in the design and conduct of surgical trials in children:

- Develop clear and accessible parent information, with input from parents of other children who have 'walked in their shoes'
- Provide the information sheet well in advance of their planned surgery, eg. send by post, when feasible

For peer review only

- Signpost to educational resources, such as the NHLBI's online *Children and Clinical Studies* (http://www.childrenandclinicalstudies.org/), that may improve understanding [25]
- Explain any potential risks associated with the trial, but separate these from the risks of the operation
- Minimise additional procedures eg. take blood only from indwelling lines
- Highlight the potential benefits of the trial, without overstating any personal benefit
- Build on the parents' trust in their clinical team, with a close and visible working relationship with trialists, ensuring that clinicians are well informed to discuss the trial with parents if asked.

For peer review only

Strengths and limitations

The strengths of this study include the extensive use of PPI, with a focus group discussion to shape the topic guide for the interviews, young person input to the protocol, and parental review of the study documents to improve clarity and readability. We allowed a run-in period for the trial so the impact of any familiarisation phase would be minimised. Most parents who were approached for interview were happy to take part, enabling us to reach data saturation for consenting parents in a timely manner, within the duration of trial recruitment. Interviews were arranged at the convenience of parents, either in person at home, in the clinic or by telephone, to facilitate their participation and were conducted by a senior nurse researcher, skilled in conducting qualitative interviews but independent of their clinical team to reduce the risk of confirmation bias or a halo effect. The BRICC trial was a suitable vehicle to explore parents' perspectives on clinical research as the intervention (remote ischaemic preconditioning) presents minimal risk, the surgery is performed electively, and the operations included have a low predicted mortality (STAT categories 1-2) [28]. The limitations include only two interviews with parents who declined the trial, providing limited insight into the actual reasons for declining. This was a consequence of both the high overall

For peer review only - http://bmjopen.bmj.com/site/about/guidelines.xhtml

consent rate in the trial, approximately 85%, limiting the pool of decliners, and that most parents who declined the trial also declined to be interviewed; these parents are a seldom heard group who may have seen the approach for interview as intrusive or seeming to question their decision not to take part.

Conclusions

Parents of children undergoing cardiac surgery attach value to clinical research and are supportive of clinical trials. The most important factors that influence decision-making on whether to allow their child to take part are perceived risk, potential benefit either to their child or others, information and timing of approach, and trust in their clinical team. Trial recruitment and retention may be improved by addressing communication and information needs, particularly surrounding potential risk, and improving collaborative working with clinicians. Our findings contribute to knowledge surrounding the acceptability of research in children undergoing surgery and should be used to inform the design and conduct of future clinical trials.

For peer review only

Acknowledgements: We thank Dr Jonathan Ives, University of Bristol for advice on development of the protocol and Helen Winmill, Jenna Spry & colleagues in PICU Research nursing team for assistance in recruitment. We are grateful to the Young Person's Steering Group for advice on the protocol, the trustees of *Young at Heart* for feedback on the study documents, and Sandra Ramsey & Martina Ponsonby for help with the focus group. We thank our surgical colleagues Ms Natasha Khan, Mr Phil Botha and formerly Prof David Barron, for their contribution to the BRICC trial, and The Transcription Company for transcription of the digital recordings. We are most grateful to the parents who either participated in the focus group or were interviewed for this study.

Author contributions: NED: conceptualisation, data curation, formal analysis, funding acquisition, methodology, resources, writing original draft, writing review & editing. JCM: data curation, formal analysis, investigation, methodology, project administration, writing original draft, writing review & editing. CJT: conceptualisation, methodology, writing review & editing. TJJ: conceptualisation, funding acquisition, writing review & editing. ACL: data curation, formal analysis, methodology, supervision, writing review & editing.

Funding: This work was supported by a grant from the Birmingham Children's Hospital Research Foundation [BCHRF442] and an Intermediate Clinical Research Fellowship from the British Heart Foundation [FS/15/49/31612] awarded to Nigel Drury. Lay review of study documents was funded by a PPI Bursary [RDS/WM-1318] from the National Institute for Health Research (NIHR) West Midlands Research Design Service. Julie Menzies is an NIHR 70@70 Senior Nurse and Midwife Research Leader, and Clare Taylor is funded by an NIHR Academic Clinical Lectureship. The Institute of Cardiovascular Sciences is supported by an Accelerator Award from the British Heart Foundation [AA/18/2/34218]. Neither the sponsor nor funders had any role in the design of this study, data collection, interpretation, or decision to submit the results for publication.

For peer review only - http://bmjopen.bmj.com/site/about/guidelines.xhtml

Disclaimer: The views expressed are those of the authors and not necessarily those of the NHS, Department of Health, NIHR, Birmingham Women's and Children's NHS Foundation Trust, British Heart Foundation, University of Birmingham, or University of Oxford.

Competing interests: Clare Taylor reports personal fees from Novartis and Vifor, outside the submitted work. No other conflicts of interest declared.

Ethics approval: West Midlands-Solihull NHS Research Ethics Committee (17/WM/088) on 28 April 2017.

Data sharing statement: The original data are available from the corresponding author upon reasonable request.

For peer review only

Figure legends

Figure 1. Participant flow diagram.

Figure 2. Factors affecting parental decision-making.

For peer review only - http://bmjopen.bmj.com/site/about/guidelines.xhtml

Table 1. Child and interview participant demographics.

**Children**	n = 23
Age at surgery, median (IQR) (months)	9 (5-14)
Congenital heart disease, n (%)	
Tetralogy of Fallot	12 (52)
Ventricular septal defect	11 (48)
Hospital length of stay post-op, median (IQR) (d)	6 (5-8)
Siblings, n (%)	
None	11 (48)
One	5 (22)
Two or more	7 (30)

**Interview participants**	n = 26
Relationship to child, n (%)	
Mother	21 (81)
Father	5 (19)
Age, n (%)	
<25 years	4 (15)
25-34 years	13 (50)
>35 years	9 (35)
Ethnicity, n (%)	
Caucasian	19 (77)
South Asian	4 (15)
Black	1 (4)
Other	1 (4)
Interviewees, n (%)	
One parent	23 (88)
Both parents	3 (12)

For peer review only

REFERENCES

1. National Institute for Cardiovascular Outcomes Research, UCL. Congenital Heart Disease. https://nicor4.nicor.org.uk/CHD/an_paeds.nsf/vwContent/home [accessed March 3, 2020]

2. Drury NE, Patel AJ, Oswald NK et al. Randomized controlled trials in children's heart surgery in the 21st century: a systematic review. *Eur J Cardiothorac Surg* 2018;53:724-31.

3. Pritchard-Jones K, Dixon-Woods M, Naafs-Wilstra M, Valsecchi MG. Improving recruitment to clinical trials for cancer in children. *Lancet Oncol* 2008;9:392-9.

4. Caldwell PH, Butow PN, Craig JC. Parents' attitudes to children's participation in randomised controlled trials. *J Pediatr* 2003;142:554-9.

5. Knox CA, Burkhart PV. Issues related to children participating in clinical research. *J Pediatr Nurs* 2007;22:310-8.

6. Shilling V, Young B. How do parents experience being asked to enter a child in a randomised controlled trial? *BMC Medical Ethics* 2009;10:1.

7. Taylor CM. Interviewing. In: Holloway I, ed. *Qualitative research in health care*. Maidenhead: Open University Press, 2005, p39-55.

8. Drury NE, Bi R, Woolley RL, Stickley J, Morris KP, Montgomerie J, van Doorn C, Dunn WB, Madhani M, Ives NJ, Kirchhof P, Jones TJ. The Bilateral Remote Ischaemic Conditioning in Children (BRICC) trial: protocol for a two-centre, double-blind, randomised controlled trial in young children undergoing cardiac surgery. *BMJ Open* 2020 [in press], doi:10.1136/bmjopen-2020-042176.

9. Kitzinger J. Focus group research: using group dynamics to explore perceptions, experiences and understandings. In: Holloway I, ed. *Qualitative research in health care*. Maidenhead: Open University Press, 2005, p56-70.

For peer review only - http://bmjopen.bmj.com/site/about/guidelines.xhtml

10. Clarke V, Braun V. *Successful Qualitative Research A Practical Guide for Beginners.* London: Sage Publications, 2013.

11. Hearps SJ, McCarthy MC, Muscara F, Hearps SJ, Burke K, Jones B et al. Psychological risk in families of infants undergoing surgery for serious congenital heart disease. *Cardiol Young* 2014;24:632-9.

12. Mason SA, Allmark PJ. Obtaining informed consent to neonatal randomised controlled trials: interviews with parents and clinicians in the Euricon study. *Lancet* 2000;356:2045-51.

13. Hoehn KS, Wernovsky G, Rychik J, Gaynor JW, Spray TL, Feudtner C et al. What factors are important to parents making decisions about neonatal research? *Arch Dis Child Fetal Neonatal Ed* 2005;90:F267-9.

14. Nathan AT, Hoehn KS, Ittenbach RF, Gaynor JW, Nicolson S, Wernovsky G et al. Assessment of parental decision-making in neonatal cardiac research: a pilot study. *J Med Ethics* 2010;36:106-10.

15. Hoffman TM, Taeed R, Niles JP, McMillin MA, Perkins LA, Feltes TF. Parental factors impacting the enrolment of children in cardiac critical care clinical trials. *Pediatr Cardiol* 2007;28:167-71.

16. Kupst MJ, Patenaude AF, Walco GA, Sterling C. Clinical trials in pediatric cancer: parental perspectives on informed consent. *J Pediatr Hematol Oncol* 2003;25:787-90.

17. Menon K, Ward R, Canadian Critical Care Trials Group. A study of consent for participation in a non-therapeutic study in the pediatric intensive care population. *J Med Ethics* 2014;40:123-6.

18. Errington J, Malik G, Evans J, Baston J, Parry A, Price L et al. Investigating the Experiences of Childhood Cancer Patients and Parents Participating in Optional Nontherapeutic Clinical Research Studies in the UK. *Pediatr Blood Cancer* 2016;63:1193-7.

For peer review only - http://bmjopen.bmj.com/site/about/guidelines.xhtml

For peer review only

19. Hoberman A, Shaikh N, Bhatnagar S, Haralam MA, Kearney DH, Colborn DK et al. Factors that influence parental decisions to participate in clinical research: consenters vs nonconsenters. *JAMA Pediatr* 2013;167:561-6.

20. Wilman E, Megone C, Oliver S, Duley L, Gyte G, Wright JM. The ethical issues regarding consent to clinical trials with pre-term or sick neonates: a systematic review (framework synthesis) of the empirical research. *Trials* 2015;16:502.

21. Tromp K, Zwaan CM, van de Vathorst S. Motivations of children and their parents to participate in drug research: a systematic review. *Eur J Pediatr* 2016;175:599-612.

22. Tait AR, Voepel-Lewis T, Malviya S. Participation of children in clinical research: factors that influence a parent's decision to consent. *Anesthesiology* 2003;99:819-25.

23. Knapp P, Raynor DK, Silcock J, Parkinson B. Can user testing of a clinical trial patient information sheet make it fit-for-purpose? – a randomized controlled trial. *BMC Med* 2011;9:89.

24. Pemberton VL, Kaltman JR, Pearson GD. Children and clinical studies: The National Heart, Lung, and Blood Institute's new multimedia resource for pediatric research. *J Am Coll Cardiol* 2009;54:502-4.

25. Marceau LD, Welch LC, Pemberton VL, Pearson GD. Educating Parents About Pediatric Research: Children and Clinical Studies Website Qualitative Evaluation. *Qual Health Res* 2016;26:1114-22.

26. Menon K, Ward RE, Gaboury I, Thomas M, Joffe A, Burns K et al. Factors affecting consent in pediatric critical care research. *Intensive Care Med* 2012;38:153-9.

27. Ohye RG, Sleeper LA, Mahony L, Newburger JW, Pearson GD, Lu M et al. Comparison of shunt type in the Norwood procedure for single ventricle lesions. *N Engl J Med* 2010;362:1980-92.

For peer review only - http://bmjopen.bmj.com/site/about/guidelines.xhtml

28. O'Brien SM, Clarke DR, Jacobs JP, Jacobs ML, Lacour-Gayet FG, Pizarro C et al. An empirically based tool for analyzing mortality associated with congenital heart surgery. *J Thorac Cardiovasc Surg* 2009;138:1139-53.

For peer review only

For peer review only - http://bmjopen.bmj.com/site/about/guidelines.xhtml

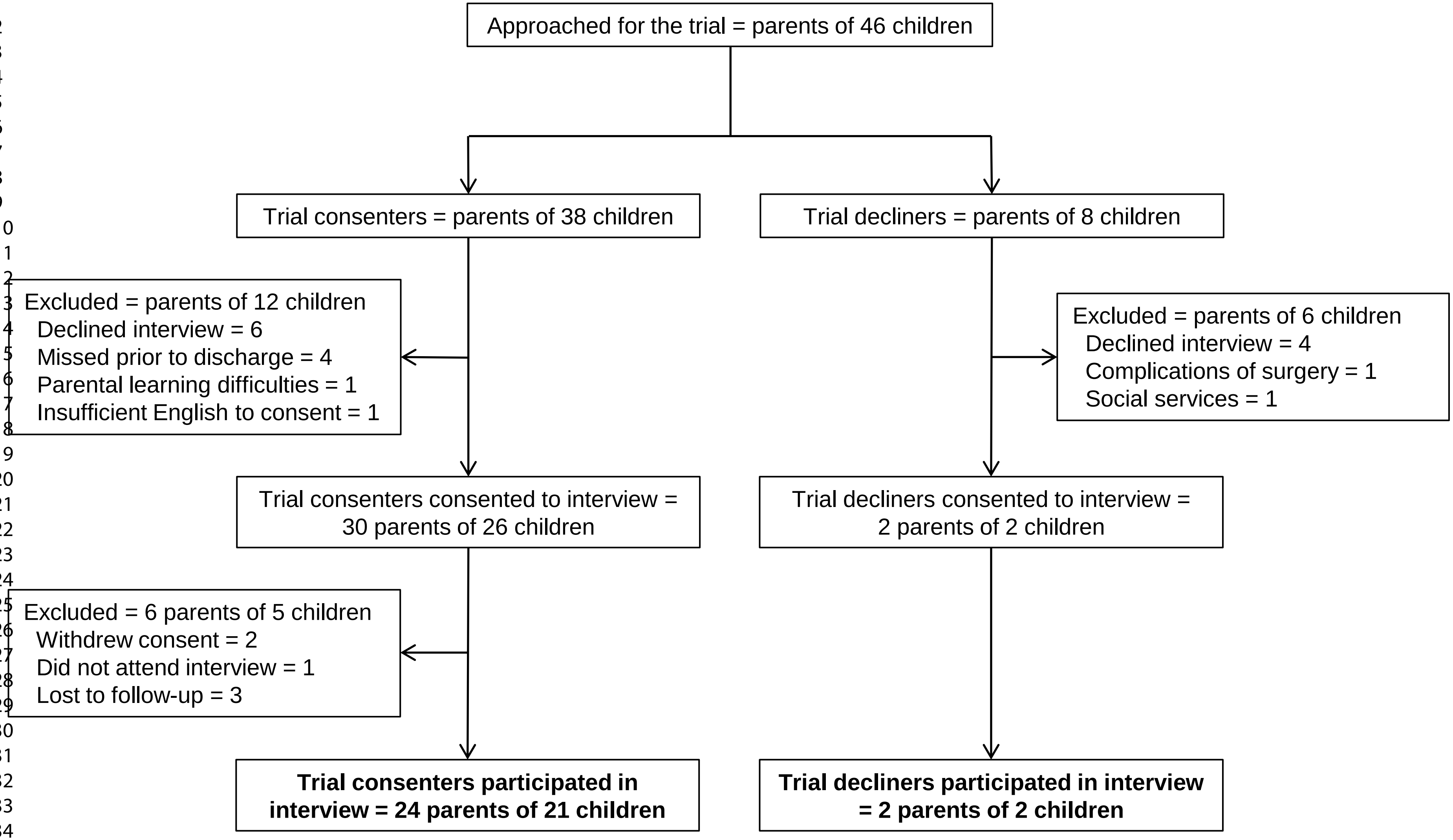
Approached for the trial = parents of 46 children
Trial consenters = parents of 38 children
Trial decliners = parents of 8 children
Excluded = parents of 12 children
Declined interview = 6
Missed prior to discharge = 4
Parental learning difficulties = 1
Insufficient English to consent = 1
Excluded = parents of 6 children
Declined interview = 4
Complications of surgery = 1
Social services = 1
Trial consenters consented to interview = 30 parents of 26 children
Trial decliners consented to interview = 2 parents of 2 children
Excluded = 6 parents of 5 children
Withdrew consent = 2
Did not attend interview = 1
Lost to follow-up = 3
Trial consenters participated in interview = 24 parents of 21 children
Trial decliners participated in interview = 2 parents of 2 children

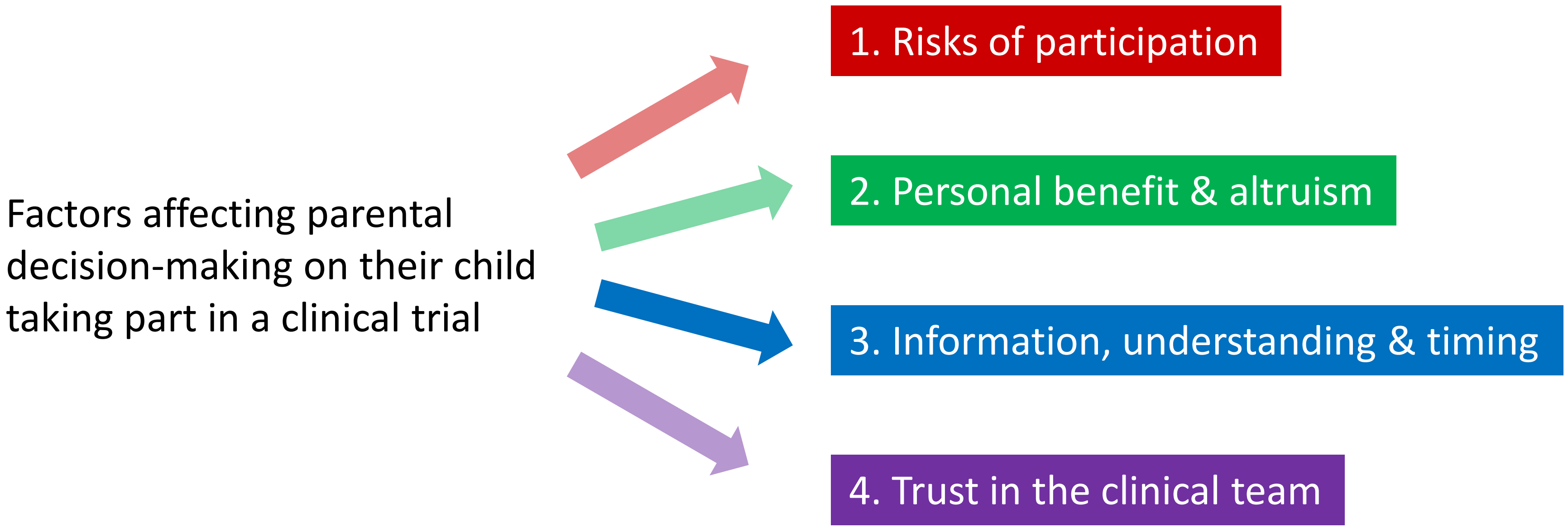
Factors affecting parental decision-making on their child taking part in a clinical trial
1. Risks of participation
2. Personal benefit & altruism
3. Information, understanding & timing
4. Trust in the clinical team

Standards for Reporting Qualitative Research (SRQR)

O'Brien B.C., Harris, I.B., Beckman, T.J., Reed, D.A., & Cook, D.A. (2014). Standards for reporting qualitative research: a synthesis of recommendations. *Academic Medicine, 89(9)*, 1245-1251.

No. Topic	Item	Page
**Title and abstract**		
S1 Title	Concise description of the nature and topic of the study identifying the study as qualitative or indicating the approach (e.g., ethnography, grounded theory) or data collection methods (e.g., interview, focus group) is recommended	1
S2 Abstract	Summary of key elements of the study using the abstract format of the intended publication; typically includes objective, methods, results, and conclusions	2
**Introduction**		
S3 Problem formulation	Description and significance of the problem/phenomenon studied; review of relevant theory and empirical work; problem statement	4
S4 Purpose or research question	Purpose of the study and specific objectives or questions	4
**Methods**		
S5 Qualitative approach and research paradigm	Qualitative approach (e.g., ethnography, grounded theory, case study, phenomenology, narrative research) and guiding theory if appropriate; identifying the research paradigm (e.g., positivist, constructivist/interpretivist) is also recommended	6
S6 Researcher characteristics and reflexivity	Researchers' characteristics that may influence the research, including personal attributes, qualifications/experience, relationship with participants, assumptions, or presuppositions; potential or actual interaction between researchers' characteristics and the research questions, approach, methods, results, or transferability	6, 17
S7 Context	Setting/site and salient contextual factors; rationale[a]	5
S8 Sampling strategy	How and why research participants, documents, or events were selected; criteria for deciding when no further sampling was necessary (e.g., sampling saturation); rationale[a]	5-6
S9 Ethical issues pertaining to human subjects	Documentation of approval by an appropriate ethics review board and participant consent, or explanation for lack thereof; other confidentiality and data security issues	5-6
S10 Data collection methods	Types of data collected; details of data collection procedures including (as appropriate) start and stop dates of data collection and analysis, iterative process, triangulation of sources/methods, and modification of procedures in response to evolving study findings; rationale[a]	5-6
S11 Data collection instruments	Description of instruments (e.g., interview guides,	6

For peer review only

and technologies	questionnaires) and devices (e.g., audio recorders) used for data collection; if/how the instrument(s) changed over the course of the study	
S12 Units of study	Number and relevant characteristics of participants, documents, or events included in the study; level of participation (could be reported in results)	8
S13 Data processing	Methods for processing data prior to and during analysis, including transcription, data entry, data management and security, verification of data integrity, data coding, and anonymization/deidentification of excerpts	6-7
S14 Data analysis	Process by which inferences, themes, etc., were identified and developed, including researchers involved in data analysis; usually references a specific paradigm or approach; rationale[a]	6-8
S15 Techniques to enhance trustworthiness	Techniques to enhance trustworthiness and credibility of data analysis (e.g., member checking, audit trail, triangulation); rationale[a]	6
**Results/Findings**		
S16 Synthesis and interpretation	Main findings (e.g., interpretations, inferences, and themes); might include development of a theory or model, or integration with prior research or theory	8-12
S17 Links to empirical data	Evidence (e.g., quotes, field notes, text excerpts, photographs) to substantiate analytic findings	8-12
**Discussion**		
S18 Integration with prior work, implications, transferability, and contribution(s) to the field	Short summary of main findings; explanation of how findings and conclusions connect to, support, elaborate on, or challenge conclusions of earlier scholarship; discussion of scope of application/generalizability; identification of unique contribution(s) to scholarship in a discipline or field	13-17
S19 Limitations	Trustworthiness and limitations of findings	17-18
**Other**		
S20 Conflicts of interest	Potential sources of influence or perceived influence on study conduct and conclusions; how these were managed	20
S21 Funding	Sources of funding and other support; role of funders in data collection, interpretation, and reporting	19

[a]The rationale should briefly discuss the justification for choosing that theory, approach, method, or technique rather than other options available, the assumptions and limitations implicit in those choices, and how those choices influence study conclusions and transferability. As appropriate, the rationale for several items might be discussed together.

For peer review only

BMJ Open

Understanding parents' decision-making on participation in clinical trials in children's heart surgery: a qualitative study

Journal:	*BMJ Open*
Manuscript ID	bmjopen-2020-044896.R1
Article Type:	Original research
Date Submitted by the Author:	23-Nov-2020
Complete List of Authors:	Drury, Nigel ; University of Birmingham, Institute of Cardiovascular Sciences; Birmingham Children's Hospital NHS Foundation Trust, Paediatric Cardiac Surgery<br>Menzies, Julie; Birmingham Children's Hospital, Paediatric Intensive Care<br>Taylor, Clare; University of Oxford, Nuffield Department of Primary Care Health Sciences<br>Jones, Timothy; Birmingham Children's Hospital, Paediatric Cardiac Surgery; University of Birmingham, Institute of Cardiovascular Sciences<br>Lavis, Anna; University of Birmingham, Institute of Applied Health Research
**Primary Subject Heading**:	Surgery
Secondary Subject Heading:	Cardiovascular medicine, Paediatrics
Keywords:	Paediatric cardiac surgery < PAEDIATRIC SURGERY, QUALITATIVE RESEARCH, Clinical trials < THERAPEUTICS

SCHOLARONE™
Manuscripts

I, the Submitting Author has the right to grant and does grant on behalf of all authors of the Work (as defined in the below author licence), an exclusive licence and/or a non-exclusive licence for contributions from authors who are: i) UK Crown employees; ii) where BMJ has agreed a CC-BY licence shall apply, and/or iii) in accordance with the terms applicable for US Federal Government officers or employees acting as part of their official duties; on a worldwide, perpetual, irrevocable, royalty-free basis to BMJ Publishing Group Ltd ("BMJ") its licensees and where the relevant Journal is co-owned by BMJ to the co-owners of the Journal, to publish the Work in this journal and any other BMJ products and to exploit all rights, as set out in our licence.

The Submitting Author accepts and understands that any supply made under these terms is made by BMJ to the Submitting Author unless you are acting as an employee on behalf of your employer or a postgraduate student of an affiliated institution which is paying any applicable article publishing charge ("APC") for Open Access articles. Where the Submitting Author wishes to make the Work available on an Open Access basis (and intends to pay the relevant APC), the terms of reuse of such Open Access shall be governed by a Creative Commons licence – details of these licences and which Creative Commons licence will apply to this Work are set out in our licence referred to above.

Other than as permitted in any relevant BMJ Author's Self Archiving Policies, I confirm this Work has not been accepted for publication elsewhere, is not being considered for publication elsewhere and does not duplicate material already published. I confirm all authors consent to publication of this Work and authorise the granting of this licence.

For peer review only

Understanding parents' decision-making on participation in clinical trials in children's heart surgery: a qualitative study

Nigel E Drury [1,3]*, Julie C Menzies [2]*, Clare J Taylor [5], Timothy J Jones [1,3], Anna C Lavis [4]

[1] Department of Paediatric Cardiac Surgery, Birmingham Children's Hospital, Birmingham, UK; [2] Department of Paediatric Intensive Care, Birmingham Children's Hospital, Birmingham, UK; [3] Institute of Cardiovascular Sciences, University of Birmingham, Birmingham, UK; [4] Institute of Applied Health Research, University of Birmingham, Birmingham, UK; [5] Nuffield Department of Primary Care Health Sciences, University of Oxford, Oxford, UK.

* Nigel Drury and Julie Menzies contributed equally to this work.

Correspondence: Mr Nigel Drury, Department of Paediatric Cardiac Surgery, Birmingham Children's Hospital, Steelhouse Lane, Birmingham, B4 6NH, UK.

Tel: +44 121 333 8731, fax: +44 121 333 9441, email: n.e.drury@bham.ac.uk

Word count: 4059 (excluding title page, abstract, references, figures, and table)

Number of figures: 2

Number of tables: 1

Keywords: Paediatric cardiac surgery, parents, qualitative study, clinical trials, decision-making.

For peer review only - http://bmjopen.bmj.com/site/about/guidelines.xhtml

Abstract

Objectives: Few children undergoing heart surgery are recruited to clinical trials and little is known about the views and attitudes of parents towards trials. This study explored parents' perspectives on decision-making about their child's participation in a clinical trial during their elective cardiac surgery.

Design: Qualitative interview study.

Setting: Single centre sub-study of a multi-centre, double-blind, randomised controlled trial to investigate the effects of remote ischaemic preconditioning in children undergoing cardiac surgery.

Participants: Parents of children approached to participate in the trial, both consenters and decliners.

Methods: Semi-structured interviews were conducted face-to-face or by telephone following discharge, digitally audio-recorded, transcribed, and thematically analysed.

Results: Of 46 patients approached for the trial, 24 consenting and 2 declining parents agreed to participate in an interview (21 mothers, 5 fathers). Parental decision-making about research was influenced by 1) potential risks or additional procedures, 2) personal benefit and altruism for the 'cardiac community'; 3) information, preparation, timing and approach; and 4) trust in the clinical team and collaboration with researchers. All of these were placed within the context of their understanding of the trial and knowledge of research.

Conclusions: Parents of children undergoing cardiac surgery attach value to clinical research and are supportive of clinical trials when there is no or minimal perceived additional risk. These findings enhance our understanding of the factors that influence parents' decision-making and should be used to inform the design and conduct of future paediatric surgical trials.

Abstract word count: 238

For peer review only - http://bmjopen.bmj.com/site/about/guidelines.xhtml

Strengths and limitations of this study

- This qualitative sub-study aimed to identify the most important issues that influence parents' decision-making on whether to allow their child to take part in a surgical trial, to improve the design and conduct of future trials.
- The interview topic guide, protocol, and study documents were developed with extensive patient and public involvement.
- The clinical trial was a suitable vehicle to explore parents' perspectives on research as the surgery was elective with low predicted mortality and the trial intervention presented minimal additional risk.
- The study reached data saturation for parents who consented to the trial but was limited by the low number of parents who agreed to be interviewed after declining their child's participation.
- Parents were recruited from a single large paediatric cardiac surgical centre in the United Kingdom which may limit generalisability.

For peer review only

For peer review only - http://bmjopen.bmj.com/site/about/guidelines.xhtml

INTRODUCTION

Randomised controlled trials are the accepted gold standard to evaluate the efficacy of treatments, promote evidence-based practice and improve the quality of clinical care. However, of the approximately 4,500 children who undergo surgery for congenital heart disease annually in the United Kingdom [1], less than 1% have been recruited to cardiac surgical trials, all of which have been small, single-centre, phase II trials [2]; in contrast, over 70% of children diagnosed with cancer are enrolled into national or international late phase trials [3] As a congenital heart disease community, we have a responsibility to conduct well-designed, multi-centre trials to answer key questions to improve the outcomes of surgery for children and their families [2].

Recruitment to paediatric trials is recognised to be challenging [4,5] but can be improved by understanding the factors that are important to parents when considering whether to allow their child to take part. The role of parents in this decision-making is complex, balancing the perceived risks and benefits of taking part [6]. However, little is known about the views and attitudes of the parents of children undergoing cardiac surgery towards involvement in research and specifically clinical trials; by understanding parents' perspectives, we can support their decision-making by improving the design and conduct of future trials. This knowledge is useful for enhancing families' experiences of trial participation and increasing recruitment [6], thereby expanding the evidence-base to guide treatment and improve patient outcomes. Qualitative studies provide participants' experiences in their own words, allowing exploration of the meanings they attribute to them, which is crucial to getting beyond assumptions about what matters in the processes of decision-making [7]. We conducted interviews with the parents of children approached to participate in a low-risk, double-blind, randomised controlled trial to explore their perspectives on research involving their child, with the aim to better understand the factors that influence their decision whether or not to participate in a clinical trial.

For peer review only - http://bmjopen.bmj.com/site/about/guidelines.xhtml

MATERIALS AND METHODS

This qualitative study was a single-centre sub-study of the Bilateral Remote Ischaemic Conditioning in Children (BRICC) trial, a multi-centre, double-blind, randomised controlled trial of remote ischaemic preconditioning in children (ISRCTN 12923441) [8]. In the trial, children aged 3 months to 3 years undergoing elective surgery for either isolated ventricular septal defect closure or tetralogy of Fallot repair were recruited. Parents were provided with the trial parent/guardian information sheet (PIS) either in the clinic/ward or sent in the post and usually given at least two weeks, but no less than 24 hours, to consider their child's participation and ask questions. Written informed consent was obtained by a Consultant, usually not the surgeon performing the operation and typically on the day before surgery. Bilateral lower limb preconditioning was performed after induction of anaesthesia but prior to sternotomy. Right atrial (additional) +/- right ventricular (when routinely resected) biopsies were obtained intraoperatively and blood samples were taken from indwelling lines during the first 24 hours after surgery. Both the clinical trial (16/WM/0309, 5 August 2016) and this qualitative sub-study (17/WM/088, 28 April 2017) were approved by the West Midlands-Solihull NHS Research Ethics Committee and sponsored by the University of Birmingham (RG_14-025 and RG_17-005, respectively).

Patient and Public Involvement (PPI): The sub-study protocol was reviewed and amended following feedback from the Clinical Research Network's Young Person's Steering Group in the West Midlands, comprising 11 young people and one parent. Four parents of children who had previously undergone cardiac surgery reviewed the sub-study PIS and consent forms to improve clarity and readability. Another eight parents were convened as a focus group (facilitated by NED and ACL) to discuss their opinions, beliefs, concerns and expectations of research in children's heart surgery [9], and this was used to develop an interview topic guide.

For peer review only - http://bmjopen.bmj.com/site/about/guidelines.xhtml

Participants: Parents of children approached to participate in the trial, both those who consented and those who declined, were eligible to be interviewed. Potential participants were excluded if their child had experienced a serious adverse event during or immediately after surgery to avoid further distress, including death, extracorporeal life support or further surgery in the early post-operative period, or if their level of English was insufficient to participate in the interview process. Recruitment began six months after starting the trial to allow time for the healthcare professionals to become familiar with the trial processes. Subsequently, all eligible parents were approached to participate, and it was estimated that the parents of 20-30 children would be interviewed but recruitment would stop if data saturation were reached or trial recruitment was completed.

Recruitment: Following discharge from PICU, one or both parents were approached on the ward by a research nurse. Parents were provided with the PIS and offered the opportunity to participate in an interview at a convenient time: prior to hospital discharge, face-to-face at home, at an outpatient visit, or by telephone. With written informed consent, semi-structured interviews were conducted by an experienced qualitative interviewer (JCM), previously unknown to the parents and independent of the clinical team, usually within six weeks of discharge.

Interviews: The schedule included: understanding of the trial, research knowledge, provision of information, timing of approach, acceptability, motivations for participation, types of research, and most important factors in decision-making. As subsequent member checking by participants for accuracy and resonance with their experiences was deemed impractical, participant validation was performed during the interviews by summarising, repeating or paraphrasing the participants' words [7]. Field notes were recorded to provide context, aid interpretation, and document emotions and non-verbal behaviours. Risk to participants was deemed to be minimal but in the event of distress or concern about events which had occurred, parents were signposted to their cardiac specialist nurse, General Practitioner, or the Patient Advice Liaison Service (PALS), as appropriate.

For peer review only - http://bmjopen.bmj.com/site/about/guidelines.xhtml

Analysis: Interviews were digitally audio-recorded, professionally transcribed, anonymised, and thematically analysed [10] by three researchers (JCM, NED, ACL), using NVivo 12 (QSR International, Melbourne, Australia) for data management. The initial eight interviews were coded by all researchers independently, the coding structure compared to ensure consistency and a common coding scheme developed. The remaining interviews were allotted between the three researchers who coded these independently and convened to generate themes and discuss deviant cases.

The first authors had full access to all the data and take responsibility for its integrity and analysis.

For peer review only

For peer review only - http://bmjopen.bmj.com/site/about/guidelines.xhtml

RESULTS

Between September 2017 and June 2019, the parents/guardians of 46 children were approached about their child's participation in the BRICC trial at the Birmingham site, of whom 38 consented to the trial and eight declined (figure 1). Interviews were conducted with 26 parents of 23 children, 24 consenting parents (of 21 children) and two declining parents (of two children); child and participant demographics are shown in table 1, with participant-level descriptions in the supplement. Seventeen (74%) interviews took place within six weeks of hospital discharge and all within three months. Data collection was stopped after interviewing 26 parents as it was agreed that data saturation had been reached for consenting parents.

Parental decision-making about whether their child should participate in cardiac surgical research was influenced by four key factors: 1) risks of participation and additional procedures; 2) personal benefit and altruism for the 'cardiac community'; 3) information, understanding and timing of approach; and 4) trust in the clinical team (figure 2). These were placed within the context of their understanding of the trial and knowledge of research. In the quotes, C indicates a consenting parent and D signifies a declining parent; additional quotes are provided in the supplement.

1. Risks of participation

Parental decision-making was influenced by the perceived level of risk for potential harm posed by the research. Some parents weighed up the risks and benefits of their child participating whilst for others, the focus on any perceived risk trumped any potential benefit; they could overlook a lack of personal benefit to help others but only if there was no risk to their own child.

For peer review only

> "*I was quite open to it because there was no risk… If there'd been a risk to her, I wouldn't have done it.*" (C10)

The trial involved additional procedures to standard care, such as blood sampling on PICU and intraoperative tissue biopsies. Parents were largely unconcerned about the additional blood samples, even though these were 'extra', because samples were taken from indwelling lines and therefore not associated with painful procedures. If this had involved additional venepuncture, parental attitudes would have changed; for many, the added potential distress to their child would be the determining factor.

Both parents who declined the trial were apprehensive about being involved in research *per se*, that it is something extra and therefore has an inherent and unnecessary risk.

> "*They wanted to know if it would make things easier with the operation but for us, we thought they were experimenting, so we didn't want it done. Just in case something went wrong whilst they did it. I know it's just a cuff but it's just one of those things.*" (D2)

Although, on reflection during the interview, one parent (D1) had changed her mind and would now be willing to take part.

2. Personal benefit and altruism

Parents often described considering personal benefit for their child in their decision-making. They recognised that whilst the trial intervention may have a direct benefit, the research may also help their child later in life, especially if they required further surgery. Several parents considered the potential benefit to any future unborn children who may also have a congenital heart defect and require surgery.

For peer review only - http://bmjopen.bmj.com/site/about/guidelines.xhtml

"*You need to have the research for the future. I now know that there's a risk that if I have another child, there could be a heart problem. Now we've looked back on it, heart problems actually run in our family… research may help my baby in the future.*" (C20)

Families felt a strong sense of being part of a community affected by congenital heart disease, particularly whilst in hospital. Many reflected that they were benefitting from previous parents' participation in research and that they could contribute to improving surgery and outcomes for future families. They were positive about how participating in the trial made them feel and satisfaction that their child had contributed to research. Parents who had declined the trial also recognised that participation in research may benefit other children but focused on the needs of their own child.

"*Obviously, it's not like you don't care about anyone else's child but you know when you are going through it it's like you want everything the best for them.*" (D2)

3. Information, understanding and timing

Parents described the turmoil of getting their heads around the cardiac diagnosis, what the surgery involved and how it would impact on them and their child, and it was against this background that decisions about trial participation were made. The trial PIS was written with PPI input and most parents found the quantity and complexity of information to be appropriate.

"*It was explained well, it wasn't full of, you know, big words that you don't understand and equally you know, yeah I do feel it was aimed at the right audience really.*" (C16)

However, there were often misconceptions about the trial, the intervention and how it related to the operation. There was a lack of confidence in, and some misunderstanding of, research terminology such as 'randomisation', 'blinding' and 'placebo'. Misconceptions involved a

For peer review only - http://bmjopen.bmj.com/site/about/guidelines.xhtml

belief that their child was ‘chosen’ for the study or not understanding that the study had set inclusion and exclusion criteria.

Most parents had been sent the information by post to give them time to consider their child’s involvement and discuss with family members; most found this approach agreeable and valued the time to read and reflect.

> “*We probably didn’t read it as well as we could have, but we did screenshot the information and send it to family members and asked them their opinion, so they had time and opportunity to read it without the emotion that we were going through.*” (C8)

Having had the opportunity to ask questions was also important, either in the clinic or on admission to the ward. When asked about how they would feel if they had to make an urgent decision, perhaps if their child was on PICU or the surgery was imminent, there was much greater apprehension.

> “*I think the time issue is very important for people. If it was an emergency and their child is having surgery, they probably haven't digested that and then to receive notification about this it might be overwhelming for some people and it might have been overwhelming for us too, really.*” (C6)

The key reason given by both parents who declined the trial was that they felt overwhelmed by their situation, with the forthcoming operation and their responsibilities for the rest of their families; they saw the research as an additional burden on their time, energy and emotions.

4. Trust in the clinical team

Families had a high level of confidence and trust in their clinical caregivers; the interviews were full of positivity about their surgeon, the wider clinical team and the National Health Service (NHS). Positive relationships between researchers and clinicians were associated with favourable perceptions of research and acceptability. Even when they did not

For peer review only - http://bmjopen.bmj.com/site/about/guidelines.xhtml

understand the research or concepts such as randomisation, as noted above, some parents felt that their understanding, or lack of, was not important, because of their trust that the clinical team would not do anything to harm their child. On the one hand, this meant that they did not mind if they were blinded:

> "*It doesn't really bother me to be fair because obviously you know what you're doing, and you know what…*" (C18 father) "*He's in the right hands and we trust them completely.*" (C18 mother)

whilst on the other, it suggests that there is some misunderstanding about the role of research and its relationship with clinical practice. This links back to the concepts of personal risk and benefit, in suggesting that parents may not perceive the depersonalised aspects of research, such as randomisation, instead trusting that even in the context of a trial, the surgeon will 'choose' to do what is 'best' for their child.

For peer review only

For peer review only - http://bmjopen.bmj.com/site/about/guidelines.xhtml

DISCUSSION

The diagnosis of congenital heart disease and the realisation that their child requires surgery is a particularly stressful time for parents [11]. In other conditions, the seriousness of the child's disease, risk of the intervention and urgency of participation have been identified as important influences on how parents experience recruitment, their sense of vulnerability and the success of communication [12]; however, few studies have explored parents' perspectives on clinical trials involving their child undergoing heart surgery. Hoehn et al analysed the unsolicited comments of parents of neonates undergoing cardiac surgery in Philadelphia, PA regarding their reasons for agreeing or declining to participate in research; the most common reasons were societal benefit, individual benefit for their child and perception that it posed no harm, although parents also expressed concern about the risk of the study and anti-experimentation views [13]. The same group evaluated parental decision-making using a competence assessment tool and found that despite the stress of surgery, parents were able to understand study-specific information and make informed decisions on their neonate's participation in research [14]. Finally, Hoffman and colleagues surveyed parents of children admitted for elective cardiac surgery in Columbus, OH and found that 91% thought that clinical trials would improve the quality of care whilst 74% believed that their child may receive direct benefit from enrolling in a trial [15]. In the present study, we explored the factors that influence parents' decision-making on their child's participation in a cardiac surgical trial and identified four key aspects relating to perceived risk, potential benefit to their child or others, information and timing of approach, and trust in the clinical team.

Risk

As surrogate decision-makers, the overriding consideration of parents is to act in the best interests of their child and protect them from harm [6]. Parents often feel responsible for their

For peer review only - http://bmjopen.bmj.com/site/about/guidelines.xhtml

child's outcome in a trial and find giving consent for the child to participate much more difficult than if they were participating themselves [4]. This decision may be made more difficult by the complexity of the information and the uncertainties inherent to clinical trials, including group allocation and potential benefit from the intervention; the concepts of randomisation and clinical equipoise may be both cognitively and emotionally challenging, raising concern that their child may receive a less effective treatment [16]. The sense of responsibility for decision-making may also make parents more vulnerable to regret over making the 'wrong' decision, whether they agree to participate or not, and anticipation of regret for 'failing to protect' their child may be a major influence [6]. We found that most parents were reassured that the intervention posed no or minimal additional risk and were happy for extra blood samples to be taken if it did not negatively impact on their child. Not wanting anything extra done has been identified as a common reason for declining participation [17], but by only obtaining blood from indwelling catheters, we avoided any additional pain or distress.

Benefit

Most parents find research a positive experience and are motivated by feelings of 'doing something important' and 'giving something back' [18]. Numerous studies have identified altruism as an important factor in paediatric trials, independent of any potential personal benefit to their own child [13,19-21]. We found that parents felt motivated by a strong sense of belonging to a community affected by congenital heart disease who may benefit from the research. They also recognised that their child or their siblings may directly benefit from the research if they needed future surgery; parents of children with tetralogy of Fallot are counselled that there is a high chance of needing further surgery in early adult life so they may feel more invested in the advances that future research may bring.

For peer review only - http://bmjopen.bmj.com/site/about/guidelines.xhtml

Information, understanding and timing

Parents are more likely to allow their child to participate if they have a greater understanding of the specific study and broader trial concepts [19,22]. The use of PPI in refining the clarity and readability of written trial information has been shown to improve understanding [23] whilst educational resources, such as the *Children and Clinical Studies* programme [24], can improve parental comprehension of clinical trials and be a valuable tool to aid decision-making [25].

In the setting of elective surgery, sending out the PIS in the post provided more time for parents to consider the research and an opportunity to discuss with others, including family members and their healthcare providers [19]. Parents are more likely to take part in a trial if they feel less time pressured [22]. When asked about potentially time-critical decision-making, they were far less certain about participating, even if they were very supportive of research. Although based on projection rather than lived experience, for many, the default position would be to decline; feeling overwhelmed has been identified as the most common reason for declining participation in non-therapeutic trials on PICU [17] and parents of children undergoing cardiac surgery may be less likely to consent that other parents [26]. This suggests that recruitment involving neonates undergoing surgery in the first few days of life may be more difficult, although the 84% recruitment rate for the parents of neonates undergoing the Norwood operation in the Single Ventricle Reconstruction trial is reassuring [27]. In this group, recruitment may be optimised by prenatal trial counselling, early provision of the PIS and frequent communication.

Trust

Parents' trust in the health care professionals looking after their child may influence their decision-making [19]. In this study, parents reported immense confidence in the whole multi-disciplinary team and as found by others [15], preferred for a trial to be explained by their

For peer review only - http://bmjopen.bmj.com/site/about/guidelines.xhtml

doctor or surgeon to provide reassurance on its validity and appropriateness for their child. If this is not possible, the Principal Investigator or research coordinator should be introduced by the clinical team; a close and visible working relationship between researchers and clinicians may make recruitment more effective, building on the family's trust and respect for their healthcare provider [5] and reducing inappropriate or poorly timed approaches [20]. For some, that trust extended further, outweighing their need to understand the study, believing that the clinical team would only do 'what is best' for their child. It is therefore imperative that whilst providing information about clinical trials, the direct care team avoid explicit or inadvertent coercion which may undermine the consent process.

Declining the trial

Previous studies have identified many reasons for parents declining consent including perceived risk, pain or distress, interference with routine care, child's clinical condition, avoiding additional medications or a placebo, parental anxiety, time pressure, inadequate information or understanding, lack of importance, inconvenience, approached for too many studies, and anti-experimentalism [13,17,20,22]. Both of our decliners referred to the trial as 'just another thing to worry about' and one expressed anti-experimentation views. Identifying ways to address these issues may improve recruitment to future trials.

How can we improve clinical trials in children?

Our findings suggest several factors which should be considered in the design and conduct of surgical trials in children:

- Develop clear and accessible parent information, with input from parents of other children who have 'walked in their shoes'

For peer review only - http://bmjopen.bmj.com/site/about/guidelines.xhtml

- Provide the information sheet well in advance of their planned surgery, eg. send by post, when feasible
- Signpost to educational resources, such as the NHLBI's online *Children and Clinical Studies* (http://www.childrenandclinicalstudies.org/), that may improve understanding [25]
- Explain any potential risks associated with the trial, but separate these from the risks of the operation
- Minimise additional procedures eg. take blood only from indwelling lines
- Highlight the potential benefits of the trial, without overstating any personal benefit
- Build on the parents' trust in their clinical team, with a close and visible working relationship with trialists, ensuring that clinicians are well informed to discuss the trial with parents if asked but avoiding coercion.

Strengths and limitations

The strengths of this study include the extensive use of PPI, with a focus group discussion to shape the topic guide for the interviews, young person input to the protocol, and parental review of the study documents to improve clarity and readability. We allowed a run-in period for the trial so the impact of any familiarisation phase would be minimised. Most parents who were approached for interview were happy to take part, enabling us to reach data saturation for consenting parents in a timely manner, within the duration of trial recruitment. Interviews were arranged at the convenience of parents, either in person at home, in the clinic or by telephone, to facilitate their participation and were conducted by a senior nurse researcher, skilled in conducting qualitative interviews but independent of their clinical team to reduce the risk of confirmation bias or a halo effect. The BRICC trial was a suitable vehicle to explore parents' perspectives on clinical research as the intervention (remote ischaemic preconditioning) presents minimal risk, the surgery is performed electively, and the operations included have a low predicted mortality (STAT categories 1-2) [28]. The

For peer review only

limitations include only two interviews with parents who declined the trial, providing limited insight into the actual reasons for declining. This was a consequence of both the high overall consent rate in the trial, approximately 85%, limiting the pool of decliners, and that most parents who declined the trial also declined to be interviewed; these parents are a seldom heard group who may have seen the approach for interview as intrusive or seeming to question their decision not to take part. As interviews were conducted following a period of intense stress and may have taken place up to three months following hospital discharge, there was potential for recall bias relating to their earlier thoughts and decision-making [29]. The study was also limited to a single, high-volume paediatric cardiac surgical centre in the United Kingdom which may limit generalisability.

Conclusions

Parents of children undergoing cardiac surgery attach value to clinical research and are supportive of clinical trials. The most important factors that influence decision-making on whether to allow their child to take part are perceived risk, potential benefit either to their child or others, information and timing of approach, and trust in their clinical team. Trial recruitment and retention may be improved by addressing communication and information needs, particularly surrounding potential risk, and improving collaborative working with clinicians. Our findings contribute to knowledge surrounding the acceptability of research in children undergoing surgery and should be used to inform the design and conduct of future clinical trials.

For peer review only - http://bmjopen.bmj.com/site/about/guidelines.xhtml

Acknowledgements: We thank Dr Jonathan Ives, University of Bristol for his advice on development of the protocol and Helen Winmill, Jenna Spry & colleagues in the PICU Research nursing team for assistance in recruitment. We are grateful to the Young Person's Steering Group for advice on the protocol, the trustees of *Young at Heart* for feedback on the study documents, and Sandra Ramsey & Martina Ponsonby for help with the focus group. We thank our surgical colleagues Ms Natasha Khan, Mr Phil Botha and formerly Prof David Barron, for their contribution to the BRICC trial, and The Transcription Company for transcription of the digital recordings. We are most grateful to the parents who either participated in the focus group or were interviewed for this study.

Author contributions: NED: conceptualisation, data curation, formal analysis, funding acquisition, methodology, resources, writing original draft, writing review & editing. JCM: data curation, formal analysis, investigation, methodology, project administration, writing original draft, writing review & editing. CJT: conceptualisation, methodology, writing review & editing. TJJ: conceptualisation, funding acquisition, writing review & editing. ACL: data curation, formal analysis, methodology, supervision, writing review & editing.

Funding: This work was supported by a grant from the Birmingham Children's Hospital Research Foundation [BCHRF442] and an Intermediate Clinical Research Fellowship from the British Heart Foundation [FS/15/49/31612] awarded to Nigel Drury. Lay review of study documents was funded by a PPI Bursary [RDS/WM-1318] from the National Institute for Health Research (NIHR) West Midlands Research Design Service. Julie Menzies is an NIHR 70@70 Senior Nurse and Midwife Research Leader, and Clare Taylor is funded by an NIHR Academic Clinical Lectureship. The Institute of Cardiovascular Sciences is supported by an Accelerator Award from the British Heart Foundation [AA/18/2/34218]. Neither the sponsor nor funders had any role in the design of this study, data collection, interpretation, or decision to submit the results for publication.

For peer review only - http://bmjopen.bmj.com/site/about/guidelines.xhtml

Disclaimer: The views expressed are those of the authors and not necessarily those of the NHS, Department of Health, NIHR, Birmingham Women's and Children's NHS Foundation Trust, British Heart Foundation, University of Birmingham, or University of Oxford.

Competing interests: Clare Taylor reports personal fees from Novartis and Vifor, outside the submitted work. No other conflicts of interest declared.

Ethics approval: West Midlands-Solihull NHS Research Ethics Committee (17/WM/088) on 28 April 2017.

Data sharing statement: The original data are available from the corresponding author upon reasonable request.

Figure legends

Figure 1. Participant flow diagram.

Figure 2. Factors affecting parental decision-making.

For peer review only

Table 1. Child and interview participant demographics.

**Children**	n = 23
Age at surgery, median (IQR) (months)	9 (5-14)
Congenital heart disease, n (%)	
Tetralogy of Fallot	12 (52)
Ventricular septal defect	11 (48)
Hospital length of stay post-op, median (IQR) (d)	6 (5-8)
Siblings, n (%)	
None	11 (48)
One	5 (22)
Two or more	7 (30)

**Interview participants**	n = 26
Relationship to child, n (%)	
Mother	21 (81)
Father	5 (19)
Age, n (%)	
<25 years	4 (15)
25-34 years	13 (50)
≥35 years	9 (35)
Ethnicity, n (%)	
Caucasian	19 (77)
South Asian	4 (15)
Black	1 (4)
Other	1 (4)
Interviewees, n (%)	
One parent	23 (88)
Both parents	3 (12)

For peer review only

For peer review only - http://bmjopen.bmj.com/site/about/guidelines.xhtml

REFERENCES

1. National Institute for Cardiovascular Outcomes Research, UCL. Congenital Heart Disease. https://nicor4.nicor.org.uk/CHD/an_paeds.nsf/vwContent/home [accessed July 15, 2020]

2. Drury NE, Patel AJ, Oswald NK et al. Randomized controlled trials in children's heart surgery in the 21st century: a systematic review. *Eur J Cardiothorac Surg* 2018;53:724-31.

3. Pritchard-Jones K, Dixon-Woods M, Naafs-Wilstra M, Valsecchi MG. Improving recruitment to clinical trials for cancer in children. *Lancet Oncol* 2008;9:392-9.

4. Caldwell PH, Butow PN, Craig JC. Parents' attitudes to children's participation in randomised controlled trials. *J Pediatr* 2003;142:554-9.

5. Knox CA, Burkhart PV. Issues related to children participating in clinical research. *J Pediatr Nurs* 2007;22:310-8.

6. Shilling V, Young B. How do parents experience being asked to enter a child in a randomised controlled trial? *BMC Medical Ethics* 2009;10:1.

7. Taylor CM. Interviewing. In: Holloway I, ed. *Qualitative research in health care*. Maidenhead: Open University Press, 2005, p39-55.

8. Drury NE, Bi R, Woolley RL, Stickley J, Morris KP, Montgomerie J, van Doorn C, Dunn WB, Madhani M, Ives NJ, Kirchhof P, Jones TJ. The Bilateral Remote Ischaemic Conditioning in Children (BRICC) trial: protocol for a two-centre, double-blind, randomised controlled trial in young children undergoing cardiac surgery. *BMJ Open* 2020;10:e042176.

9. Kitzinger J. Focus group research: using group dynamics to explore perceptions, experiences and understandings. In: Holloway I, ed. *Qualitative research in health care*. Maidenhead: Open University Press, 2005, p56-70.

For peer review only - http://bmjopen.bmj.com/site/about/guidelines.xhtml

10. Clarke V, Braun V. *Successful Qualitative Research A Practical Guide for Beginners.* London: Sage Publications, 2013.

11. Hearps SJ, McCarthy MC, Muscara F, Hearps SJ, Burke K, Jones B et al. Psychological risk in families of infants undergoing surgery for serious congenital heart disease. *Cardiol Young* 2014;24:632-9.

12. Mason SA, Allmark PJ. Obtaining informed consent to neonatal randomised controlled trials: interviews with parents and clinicians in the Euricon study. *Lancet* 2000;356:2045-51.

13. Hoehn KS, Wernovsky G, Rychik J, Gaynor JW, Spray TL, Feudtner C et al. What factors are important to parents making decisions about neonatal research? *Arch Dis Child Fetal Neonatal Ed* 2005;90:F267-9.

14. Nathan AT, Hoehn KS, Ittenbach RF, Gaynor JW, Nicolson S, Wernovsky G et al. Assessment of parental decision-making in neonatal cardiac research: a pilot study. *J Med Ethics* 2010;36:106-10.

15. Hoffman TM, Taeed R, Niles JP, McMillin MA, Perkins LA, Feltes TF. Parental factors impacting the enrolment of children in cardiac critical care clinical trials. *Pediatr Cardiol* 2007;28:167-71.

16. Kupst MJ, Patenaude AF, Walco GA, Sterling C. Clinical trials in pediatric cancer: parental perspectives on informed consent. *J Pediatr Hematol Oncol* 2003;25:787-90.

17. Menon K, Ward R, Canadian Critical Care Trials Group. A study of consent for participation in a non-therapeutic study in the pediatric intensive care population. *J Med Ethics* 2014;40:123-6.

18. Errington J, Malik G, Evans J, Baston J, Parry A, Price L et al. Investigating the Experiences of Childhood Cancer Patients and Parents Participating in Optional Nontherapeutic Clinical Research Studies in the UK. *Pediatr Blood Cancer* 2016;63:1193-7.

For peer review only - http://bmjopen.bmj.com/site/about/guidelines.xhtml

19. Hoberman A, Shaikh N, Bhatnagar S, Haralam MA, Kearney DH, Colborn DK et al. Factors that influence parental decisions to participate in clinical research: consenters vs nonconsenters. *JAMA Pediatr* 2013;167:561-6.

20. Wilman E, Megone C, Oliver S, Duley L, Gyte G, Wright JM. The ethical issues regarding consent to clinical trials with pre-term or sick neonates: a systematic review (framework synthesis) of the empirical research. *Trials* 2015;16:502.

21. Tromp K, Zwaan CM, van de Vathorst S. Motivations of children and their parents to participate in drug research: a systematic review. *Eur J Pediatr* 2016;175:599-612.

22. Tait AR, Voepel-Lewis T, Malviya S. Participation of children in clinical research: factors that influence a parent's decision to consent. *Anesthesiology* 2003;99:819-25.

23. Knapp P, Raynor DK, Silcock J, Parkinson B. Can user testing of a clinical trial patient information sheet make it fit-for-purpose? – a randomized controlled trial. *BMC Med* 2011;9:89.

24. Pemberton VL, Kaltman JR, Pearson GD. Children and clinical studies: The National Heart, Lung, and Blood Institute's new multimedia resource for pediatric research. *J Am Coll Cardiol* 2009;54:502-4.

25. Marceau LD, Welch LC, Pemberton VL, Pearson GD. Educating Parents About Pediatric Research: Children and Clinical Studies Website Qualitative Evaluation. *Qual Health Res* 2016;26:1114-22.

26. Menon K, Ward RE, Gaboury I, Thomas M, Joffe A, Burns K et al. Factors affecting consent in pediatric critical care research. *Intensive Care Med* 2012;38:153-9.

27. Ohye RG, Sleeper LA, Mahony L, Newburger JW, Pearson GD, Lu M et al. Comparison of shunt type in the Norwood procedure for single ventricle lesions. *N Engl J Med* 2010;362:1980-92.

For peer review only - http://bmjopen.bmj.com/site/about/guidelines.xhtml

28. O'Brien SM, Clarke DR, Jacobs JP, Jacobs ML, Lacour-Gayet FG, Pizarro C et al. An empirically based tool for analyzing mortality associated with congenital heart surgery. *J Thorac Cardiovasc Surg* 2009;138:1139-53.

29. Gertsman S, O'Hearn K, Gibson J, Menon K. Parental understanding of research consent forms in the PICU: a pilot study. *Pediatr Crit Care Med* 2020;21:526-34.

For peer review only

For peer review only - http://bmjopen.bmj.com/site/about/guidelines.xhtml

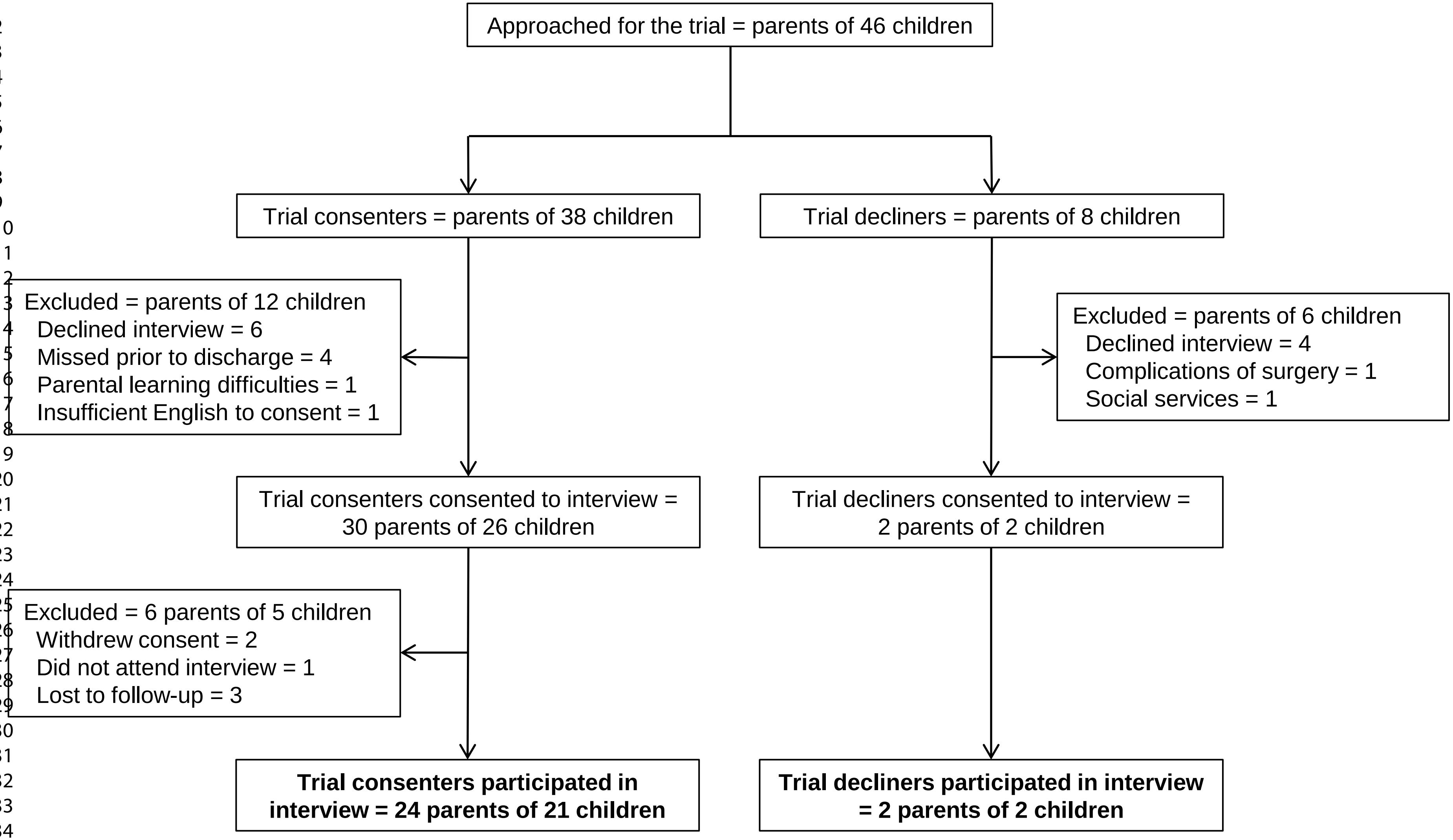
Approached for the trial = parents of 46 children
Trial consenters = parents of 38 children
Trial decliners = parents of 8 children
Excluded = parents of 12 children
Declined interview = 6
Missed prior to discharge = 4
Parental learning difficulties = 1
Insufficient English to consent = 1
Excluded = parents of 6 children
Declined interview = 4
Complications of surgery = 1
Social services = 1
Trial consenters consented to interview = 30 parents of 26 children
Trial decliners consented to interview = 2 parents of 2 children
Excluded = 6 parents of 5 children
Withdrew consent = 2
Did not attend interview = 1
Lost to follow-up = 3
Trial consenters participated in interview = 24 parents of 21 children
Trial decliners participated in interview = 2 parents of 2 children

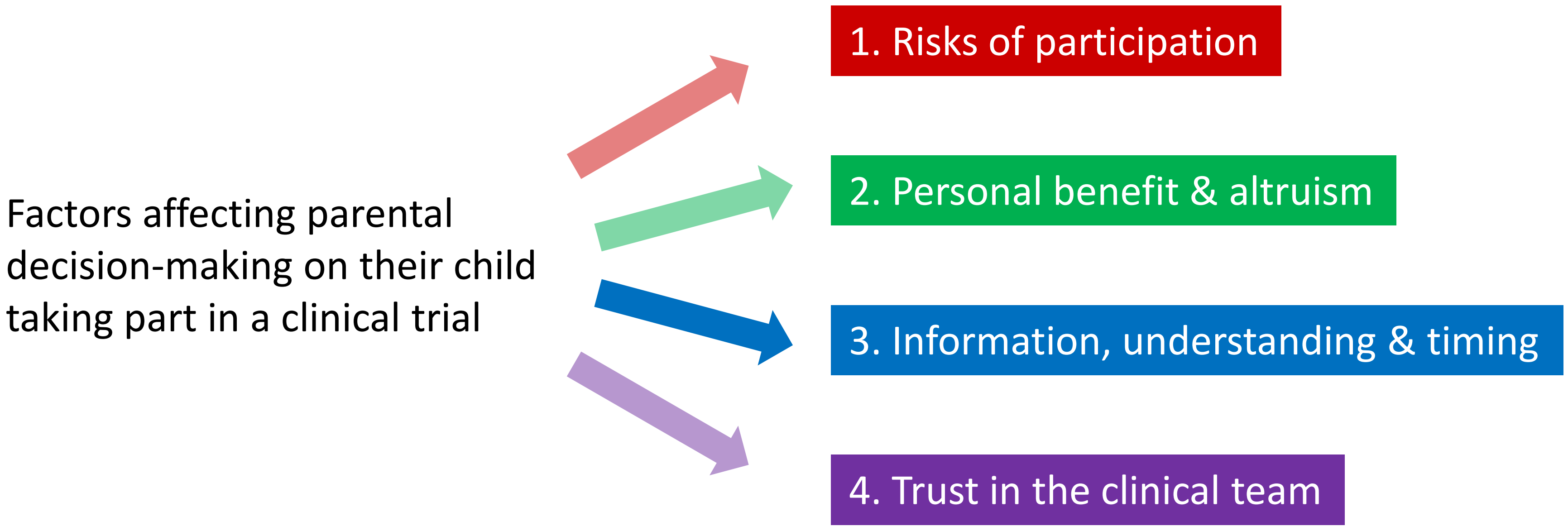
Factors affecting parental decision-making on their child taking part in a clinical trial
1. Risks of participation
2. Personal benefit & altruism
3. Information, understanding & timing
4. Trust in the clinical team

Drury NE et al. Understanding parents' decision-making on participation in clinical trials.

SUPPLEMENTARY MATERIAL

Table S1. Participant demographics

Interview	Parent(s) interviewed	Parent(s) age (years)	Parent(s) ethnicity	Type of surgery
**C1**	Mother	25-34	White British	TOF repair
**C2**	Mother	<25	White British	VSD closure
**C3**	Mother	≥35	Other	VSD closure
**C4**	Mother & Father	Both ≥35	Both South Asian	TOF repair
**C5**	Father	25-34	White British	TOF repair
**C6**	Mother	25-34	White British	TOF repair
**C7**	Mother	25-34	Black	TOF repair
**C8**	Father	25-34	White British	VSD closure
**C9**	Mother	≥35	White British	TOF repair
**C10**	Mother	25-34	White British	TOF repair
**C11**	Mother	25-34	White British	VSD closure
**C12**	Mother	25-34	South Asian	VSD closure
**C13**	Mother	≥35	White British	VSD closure
**C14**	Mother	25-34	White British	TOF repair
**C15**	Mother	25-34	White British	VSD closure
**C16**	Mother	≥35	White British	VSD closure
**C17**	Mother	<25	White British	VSD closure
**C18**	Mother & Father	Both <25	Both White British	VSD closure
**C19**	Mother	25-34	White British	TOF repair
**C20**	Mother	25-34	White British	TOF repair
**C21**	Mother & Father	Both ≥35	Both White other	VSD closure
**D1**	Mother	25-34	White British	TOF repair
**D2**	Mother	≥35	South Asian	TOF repair

C, consenting parent; D, declining parent; TOF, tetralogy of Fallot; VSD, ventricular septal defect.

For peer review only

For peer review only - http://bmjopen.bmj.com/site/about/guidelines.xhtml

Drury NE et al. Understanding parents' decision-making on participation in clinical trials.

Supplementary results: The parents/guardians of 46 children were approached about their child's participation in the trial, of whom 38 consented to the trial and eight declined, as shown in the flow diagram (figure 1). Of these, six consenting parents and four declining parents declined participation in the interview sub-study and a further six consenting (four missed prior to discharge, one had learning difficulties, and one did not speak sufficient English) and two declining (one with surgical complications and one where social services had parental responsibility) parents were not recruited. Thirty-two parents of 26 children gave consent to participate in an interview but two parents withdrew consent prior to the interview, one did not attend the interview, and three were lost to follow up. We therefore interviewed 26 parents of 23 children, 24 consenting parents (of 21 children) and two declining parents (of 2 children). These comprised 21 mothers and five fathers, with three interviewed as pairs and the remainder as one-to-one interviews; child and participant-level descriptions are shown in table S1. In the quotes, C indicates a consenting parent and D signifies a declining parent.

1. Risks of participation

Risk

"*I think again it's the risk of it, I mean he's my baby, I want to make sure that it's going to be okay, it's not going to be too risky, I wouldn't put him through anything that was going to be risky.*" (C17)

"*We were taking into consideration what the research was trying to achieve and there was actually no risk, or minimal risk.*" (C21 father)

"*I think the fact that there could've been a benefit and there not being a risk made me go for it. So, had we had known 'yes there's a benefit, there'll definitely be a benefit, but there is a risk' I wouldn't have done it. It was all about the risk factor.*" (C10)

"*If there was a risk that it could've hurt him or affected the surgery, then I wouldn't have done it.*" (C1)

"*We just really wanted to know what the risks were, whether there could be any damage to her afterwards.*" ["So, for you it was just all about the risk?"] "*Yeah definitely*" (C16)

"*Well if it had no risk, or low risk shall we say, and it gives him a better chance that you know he can do a full recovery then I can't see why not. I can't see why someone wouldn't want to do it.*" (C11)

For peer review only - http://bmjopen.bmj.com/site/about/guidelines.xhtml

"*For me personally as long as there was no risk involvement for [child], I'm more than happy for him to participate and … use his situation to help with your research. So yeah as long as there's no actual risk.*" (C19)

"*Yes absolutely, definitely, 100%. If there were any risks involved, then we wouldn't have been so - we would have to have sat down and thought about it and maybe had more conversations with whoever was leading it… It was just that there was no risk. That was the biggest thing for us.*" (C6)

"*Again, it always boils down to risk doesn't it, side effects. I think that would always be the big question for me because I wouldn't put my child under any more duress or stress than what she'd need to be.*" (C16)

Additional procedures

"*I suppose because it wasn't anything invasive, I was more inclined to say, 'Yes, just do it.' It wasn't going to affect her in any way whatsoever, and so why not? Let somebody else benefit from the results. If it was invasive, I would obviously have thought, 'Let's look into this.'*" (C20)

"*…because she already had lines in and they said, 'I just need to take a bit of blood', I said, 'Yeah, knock yourself out.* [laughter] *It's not harming her. It's not doing anything'… She didn't have a clue what was going on, bless her.*" (C20)

"*So, it was a case of them just taking extra samples or whatever the case may be, so we didn't really have to do anything.*" (C19)

"*I might have been a little bit more worried then because she was getting upset … so if that would have been the case I probably would have said no, because I wouldn't have wanted to have upset her any more, but because it was in, then yes, I was quite happy.*" (C16)

"*I think just making sure that there was no extra needles and cannulas put in, otherwise everything was fine for me to go ahead.*" (C7)

"*Yeah, I think I'd probably say no because she was traumatised when they did it. Every time they did it in there, she was traumatised.*" (C13)

"*He got all burst blood vessels from crying from the last time they pricked him. No, I wouldn't have allowed it – sorry.*" (C21 mother)

"*I'm very much that you can't progress and learn more if people don't get involved in things like research. Whereas my wife was, and me to a certain extent, was very much like she's*

For peer review only

already been through enough, without putting her through anything else while she's having surgery." (C8)

"*For weeks before we were told not to put her through anything over what she was going through already, like to really watch when she was crying, she's too sweaty. Everything was like, don't let her go. And then to have somebody say, well we're going to do this as extra, we're like well hang on, does she really need that on top of everything else?*" (C8)

Change of mind

"*I kind of feel bad now… I think I'd say yes to be honest. Now I know it's only that and not part of the operation… now I've been through it, I know obviously it's stressing and they get annoyed with everything anyway, so another thing isn't going to harm her.*" (D1)

2. Personal benefit and altruism

Personal benefit

"*It might not help my daughter this time, but research… might help her in the future.*" (C6)

"*I think reducing lifelong risks, such as damage… she might have to go and have another surgery when she's 20. I'm hoping with the research from now until then, it might not be open-heart surgery; it might be something very, very non-invasive and that's when I think the research will come into play.*" (C6)

"*Anything that we can do to help which might help her in the long run is definitely, we will do.*" (C19)

"*Our first baby, she's had tetralogy of Fallot, so automatically our percentage goes up where if we have another pregnancy that that baby could have a heart condition as well. So, you're almost helping yourself a little bit by agreeing to research on the first operation because you might be there again with the second one, do you know what I mean?*" (C10)

"*You then have a baby that actually needs something and so you need to have the research for the future. I now know that there's a risk that if I have another child, there could be a heart problem. Now we've looked back on it, heart problems actually run through the family. Obviously, your research may help my baby in the future.*" (C20)

"*If anything from the research that they did have it could have positive gains, so we weren't concerned at all about that.*" (C19)

For peer review only - http://bmjopen.bmj.com/site/about/guidelines.xhtml

"*Obviously for her to be in the position she was in and having the surgery that she was having, people must've had research studies done on them beforehand, so I just think it helps people and babies that are in our position now, in a few years. If it can make it better for them, make it a better process for them then why not do it? If there's no risk, because there was no risk to [child] through the whole process, so.*" (C10)

Altruism

"*When we left the hospital, I said, 'Oh god, we've got to go back to the real world now.' Whereas, back there, your child has got a heart problem, but so has that child and that child. You're listening to all the different scenarios, stories and medications.*" (C20)

"*You're thinking, oh my God my child's having open heart surgery you don't want nothing extra going on. But this is the main reason why we did it because he's not the first and he's never going to be the last. So, if we didn't agree for things like this, it would never get better for kids in the future.*" (C4 mother)

"*Obviously for her to be in the position she was in and having the surgery that she was having, people must've had research studies done on them beforehand, so I just think it helps people and babies that are in our position now, in a few years. If it can make it better for them, make it a better process for them then why not do it?*" (C10)

"*I feel good about it if it can help somebody. I feel good about it anyway because I feel like it's trying to help… She sure is doing some good in the world… If we can do anything to help any children, then we're willing to do it.*" (C13)

"*I feel lucky that we got a chance to take part in it. Because if the end result is something positive then that's brilliant for future babies and children that have congenital heart defects.*" (C10)

"*You feel a bit privileged really don't you when you're getting chosen to do something.*" (C2)

"*I just thought she might help other babies in the future go through a better process of having their hearts fixed, long term.*" (C10)

["So, when you agreed for [child] to take part in the study, what did you hope it would achieve?"] "*Just helping other people. I think helping other people in similar situations.*" (C7)

"*I'm happy that we did it, I'm happy that she got to take part in something that might change the way that heart patients are treated in the future.*" (C10)

For peer review only - http://bmjopen.bmj.com/site/about/guidelines.xhtml

For peer review only

"*It wasn't going to affect her in any way whatsoever, and so why not? Let somebody else benefit from the results.*" (C20)

"*Yeah quite happy to, I mean it was all for the greater good isn't it... it's actually helping future families like yourselves and their children… It's a good thing, it's the only way we're going to learn isn't it so if we can help and be part of that then yeah, we're happy to do our bit.*" (C19)

3. Information, understanding and timing

Information

"*It did give us time to ponder over it and you know look things up on the internet or speak to people. I think if we hadn't had that opportunity, I think we would have declined it.*" (C16)

"*Explain in the letter. Explain in the phone call. When you send that letter out, say, 'Please contact us if you wish to.' They can then contact you or you get the phone call in, so that you can explain the process afterwards as well. You can then say, 'Right, this is what it's actually going to entail from start to finish.*" (C20)

Understanding

"*Obviously, they were looking at, was it the blood pressure reducing up and down, so that it's not as tiring for the heart? I think I've got that from somewhere.*" (C14)

"*It's something to do with stopping blood flow to the heart before you pop it on bypass, and I can't remember… Is it to do with like how the hearts survives heart attacks, like cardiac arrests and stuff like that?*" (C10)

["Did you understand what the study was trying to find out?"] "*I can't remember that much!*" [laughs] (C7)

"*As far as I was aware it was going to, the cuffs were going to inflate and deflate and it was going to see whether something was released into her blood that was going to help the heart, that was kind of how it was explained.*" (C8)

"*Just that he was picked, I don't know really, it was just he was either going to be chosen or he wasn't for the study.*" (C2)

"*Picking people at random who've obviously all got a heart condition, but it might be something different to what* [she] *had.*" (C5)

For peer review only - http://bmjopen.bmj.com/site/about/guidelines.xhtml

Timing

"*Reading through that paperwork is the biggest thing, trying to process it because it's okay people telling you... but when you read it you think over it better yourself… The more information you get, the calmer you feel really.*" (C2)

"*I'd like to speak to the surgeons and ask more questions from them as to any concerns, rather than just getting a sheet of information because you always have extra questions, I'd want to ask a few more.*" (C19)

"*Having the time and people to help you understand what's going to happen, so you fully understand what's going to happen, because we didn't fully understand.*" (C8)

"*The Friday before she was due to go in, we came and spoke to the surgeons and we came and spoke to a nurse and that's the time when they talk you through the operation and things. So, any questions we had about that research we could have covered then.*" (C8)

"*We did ask for advice. In that respect, perhaps if it would have been rushed or an intense environment, then it would have been a bit harder for us to come to that decision.*" (C21 father)

"*I mean we had just enough time I think, we had about a week from when we got the letter to when we actually got the phone call to come in. So that wasn't too bad, that was enough time for me.*" (C17)

"*I think to have to make that decision in a short space of time, some people can, because they can cope with it, me personally I couldn't. I couldn't just make that decision because I'd just be thinking to get my child medically well and I'd probably be thinking a research study is the last thing on my mind to be dealing with.*" (C16)

"*And it's a lot to take in then when you're also thinking you're going to be having an operation, and then there's something else you're being asked to do… obviously from a parent perspective anything is unnerving anyway, your child's going through that as it is, you don't want to add anything else.*" (D1)

"*No, because it's just one of those things we don't need to worry about when you've got so much in our head as it is… when you are going through something, only you know what you are going through and at that point you can't even think straight.*" (D2)

"*I think if it was an emergency situation maybe my decision may not have been so quick or whether it would happen at all, really.*" (C6)

For peer review only - http://bmjopen.bmj.com/site/about/guidelines.xhtml

"*If you're approached just days before the operation, you might not want to do it. But if you're approached long before you're even given a date, as long as you've got it in your mind that something's taking place, I think more and more people would do it.*" (C4 mother)

"*Well, obviously when you gave the phone call it was a lot more, I'd got a bit of time to think about whether or not I want him to take part. So, with them asking me on the phone and then when we actually got there then speaking to us properly about it and trying to explain everything it was a lot more easier than somebody just coming into your room and saying, 'Well this is what we need to do, he's going for his operation in like two hours so can you decide on whether you want him to do it or not?*" (C2)

"*I think, it all goes back to time. I think if we'd had more time to read it and we were less emotional so we weren't actually in the hospital waiting and had the opportunity to speak to someone about it at the point when we came to have a look around ICU… we could have had someone sit down and just spend five minutes explaining it. Because when we talked about the heart, they got a little model out and they explained exactly what they were going to do, exactly what the risks were. I think something similar to that would have helped.*" (C8)

"*At that point I was lost* [on intensive care]*. My baby was in heart failure because he was late diagnosed and if things were different, like I said, I panic and I hit the panic button quite quickly so for a mum like me I don't think I'd be listening to anyone.*" (C3)

"*When you're sitting there and like when we first walked in and saw* [daughter] *tubed up and machines breathing for her, it really did hit home what she'd been through. But I think as the days go by and you're sitting there, I think you do become more relaxed when they start turning machines off so, if someone came to you after a couple of days saying, 'Do you mind if we just try this?*'" (C5)

"*It might have been a bit overwhelming because the research thing is an extra and sometimes, parents don't have the ability to think of these extra things when they're in an Intensive Care environment.*" (C21 mother)

"*Next to us, in PICU, was a very young baby. My wife spoke to the parents and they didn't know that their baby had a heart condition. I think they got rushed into the hospital and then it was one surgery after the other. They were quite overwhelmed. They were very receptive parents but in such a situation, I don't know how much they would factor in that there were studies going on in ICU or in the hospital.*" (C21 father)

For peer review only - http://bmjopen.bmj.com/site/about/guidelines.xhtml

For peer review only

4. Trust in the clinical team

"*We just had a great deal of confidence in Birmingham Children's* [Hospital]. *They really instilled us with so much confidence.*" (C6)

"*You just have to listen to what the doctors are telling you and put your trust in them that they know what they're doing, they're the experts.*" (C16)

"*I thought to myself that surgeons know best, if it was going to affect her in any way, I don't think they'd agree to it generally. So I guess it's just putting that trust in the surgeon, they know what's better for the patient when they open them up… so if the surgeon felt that it wasn't appropriate… they probably would have said no thank you.*" (C12)

"*You just trust the surgeons that they take as much or as little as they need to, and they do… I suppose we just put our trust in them that they're doing what they need to do.*" (C19)

"*I'll put my trust in the NHS and I know that they know what they're doing, and I wouldn't question like…*" (C18 mother)

"*If you've got these exceptional surgeons and doctors telling you, 'This is serious, but we need to do it now, ready for when she's older,' you take that information… It was purely, we didn't have to think, 'Oh, we don't want the surgery.' We didn't have to think because it was just so absolutely, 'Get her in as soon as you can,' really. I wanted it done as soon as possible.*" (C6)

"*When he came out and he was looking at her, he actually did look almost proud of his work. That fills you with confidence because you think, 'If you think that's good...' I don't know, I'm not a surgeon… When we were sat there and he looked proud of his work, I thought, 'Okay, yeah. We're good. We're alright. We're in safe hands.'* [Laughter] *It's all about being safe, isn't it?*" (C20)

For peer review only - http://bmjopen.bmj.com/site/about/guidelines.xhtml

Standards for Reporting Qualitative Research (SRQR)

O'Brien B.C., Harris, I.B., Beckman, T.J., Reed, D.A., & Cook, D.A. (2014). Standards for reporting qualitative research: a synthesis of recommendations. *Academic Medicine, 89(9)*, 1245-1251.

No. Topic	Item	Page
**Title and abstract**		
S1 Title	Concise description of the nature and topic of the study identifying the study as qualitative or indicating the approach (e.g., ethnography, grounded theory) or data collection methods (e.g., interview, focus group) is recommended	1
S2 Abstract	Summary of key elements of the study using the abstract format of the intended publication; typically includes objective, methods, results, and conclusions	2
**Introduction**		
S3 Problem formulation	Description and significance of the problem/phenomenon studied; review of relevant theory and empirical work; problem statement	4
S4 Purpose or research question	Purpose of the study and specific objectives or questions	4
**Methods**		
S5 Qualitative approach and research paradigm	Qualitative approach (e.g., ethnography, grounded theory, case study, phenomenology, narrative research) and guiding theory if appropriate; identifying the research paradigm (e.g., positivist, constructivist/interpretivist) is also recommended	6
S6 Researcher characteristics and reflexivity	Researchers' characteristics that may influence the research, including personal attributes, qualifications/experience, relationship with participants, assumptions, or presuppositions; potential or actual interaction between researchers' characteristics and the research questions, approach, methods, results, or transferability	6, 17
S7 Context	Setting/site and salient contextual factors; rationale[a]	5
S8 Sampling strategy	How and why research participants, documents, or events were selected; criteria for deciding when no further sampling was necessary (e.g., sampling saturation); rationale[a]	5-6
S9 Ethical issues pertaining to human subjects	Documentation of approval by an appropriate ethics review board and participant consent, or explanation for lack thereof; other confidentiality and data security issues	5-6
S10 Data collection methods	Types of data collected; details of data collection procedures including (as appropriate) start and stop dates of data collection and analysis, iterative process, triangulation of sources/methods, and modification of procedures in response to evolving study findings; rationale[a]	5-6
S11 Data collection instruments	Description of instruments (e.g., interview guides,	6

For peer review only

and technologies	questionnaires) and devices (e.g., audio recorders) used for data collection; if/how the instrument(s) changed over the course of the study	
S12 Units of study	Number and relevant characteristics of participants, documents, or events included in the study; level of participation (could be reported in results)	8
S13 Data processing	Methods for processing data prior to and during analysis, including transcription, data entry, data management and security, verification of data integrity, data coding, and anonymization/deidentification of excerpts	6-7
S14 Data analysis	Process by which inferences, themes, etc., were identified and developed, including researchers involved in data analysis; usually references a specific paradigm or approach; rationale[a]	6-8
S15 Techniques to enhance trustworthiness	Techniques to enhance trustworthiness and credibility of data analysis (e.g., member checking, audit trail, triangulation); rationale[a]	6
**Results/Findings**		
S16 Synthesis and interpretation	Main findings (e.g., interpretations, inferences, and themes); might include development of a theory or model, or integration with prior research or theory	8-12
S17 Links to empirical data	Evidence (e.g., quotes, field notes, text excerpts, photographs) to substantiate analytic findings	8-12
**Discussion**		
S18 Integration with prior work, implications, transferability, and contribution(s) to the field	Short summary of main findings; explanation of how findings and conclusions connect to, support, elaborate on, or challenge conclusions of earlier scholarship; discussion of scope of application/generalizability; identification of unique contribution(s) to scholarship in a discipline or field	13-17
S19 Limitations	Trustworthiness and limitations of findings	17-18
**Other**		
S20 Conflicts of interest	Potential sources of influence or perceived influence on study conduct and conclusions; how these were managed	20
S21 Funding	Sources of funding and other support; role of funders in data collection, interpretation, and reporting	19

[a]The rationale should briefly discuss the justification for choosing that theory, approach, method, or technique rather than other options available, the assumptions and limitations implicit in those choices, and how those choices influence study conclusions and transferability. As appropriate, the rationale for several items might be discussed together.

For peer review only
